# LGN loss randomizes spindle orientation and accelerates tumorigenesis in PTEN-deficient epidermis

**DOI:** 10.1091/mbc.E23-03-0111

**Published:** 2024-01-12

**Authors:** Sophie Viala, Charlotte Hadjadj, Vandana Nathan, Marie-Christine Guiot, Luke McCaffrey, Katie Cockburn, Maxime Bouchard

**Affiliations:** a Rosalind and Morris Goodman Cancer Institute and Department of Biochemistry, McGill University, Montreal H3A 1A3, Canada; b Department of Pathology, McGill University, Montreal H3A 2B4, Canada; c Gerald Bronfman Department of Oncology, McGill University, Montreal H4A 3T2, Canada; Duke University

## Abstract

Loss of cell polarity and disruption of tissue organization are key features of tumorigenesis that are intrinsically linked to spindle orientation. Epithelial tumors are often characterized by spindle orientation defects, but how these defects impact tumor formation driven by common oncogenic mutations is not fully understood. Here, we examine the role of spindle orientation in adult epidermis by deleting a key spindle regulator, LGN, in normal tissue and in a PTEN-deficient mouse model. We report that LGN deficiency in PTEN mutant epidermis leads to a threefold increase in the likelihood of developing tumors on the snout, and an over 10-fold increase in tumor burden. In this tissue, loss of LGN alone increases perpendicular and oblique divisions of epidermal basal cells, at the expense of a planar orientation of division. PTEN loss alone does not significantly affect spindle orientation in these cells, but the combined loss of PTEN and LGN fully randomizes basal spindle orientation. A subset of LGN- and PTEN-deficient animals have increased amounts of proliferative spinous cells, which may be associated with tumorigenesis. These results indicate that loss of LGN impacts spindle orientation and accelerates epidermal tumorigenesis in a PTEN-deficient mouse model.

## Abstract (executive summary)

Spindle orientation defects are often associated with epithelial tumors but how these defects impact tumor formation is not fully understood. Using complex mouse models and imaging techniques, including time lapse movies, we examine the role of spindle orientation in epidermal tumor initiation by combining the loss of LGN, a spindle regulator, and PTEN, a tumor suppressor.

We find that while homeostatic tissue can cope with spindle orientation defects, loss of both targets randomizes spindle orientation and accelerates tumor initiation in the snout.

These results suggest that altered spindle orientation can contribute to epidermal tumor initiation.

## INTRODUCTION

The skin’s outermost layer, the epidermis, is the body’s first line of defence against potential insults from the environment. It is a stratified epithelium with a high turnover sustained by a proliferative basal layer. Basal cells divide and differentiate to replace cells that are shed from the outer epidermal surface (Gonzales and Fuchs, 2017). Once they have departed from the basal layer, keratinocytes incorporate into the spinous, granular, and finally cornified layers, undergoing terminal differentiation as they move upwards. This eventually culminates in cornification, a specialized form of cell death that produces the skin’s outermost protective barrier (Matsui and Amagai, 2015; Matsui *et al.*, 2021).

Oriented cell division is a key mechanism that promotes stratification of the developing epidermis. In the embryo, basal cells orient their divisions perpendicular to the basement membrane to generate suprabasal daughter cells that contribute to the formation of the upper layers of skin (Smart, 1970; Lechler and Fuchs, 2005; Williams *et al.*, 2014). Mitotic spindle orientation is achieved by anchoring the spindle poles to the cellular cortex through an evolutionarily conserved NuMA-LGN-Gαi complex. NuMA binds to the spindle by interacting with microtubules and the microtubule-associated motor, dynein. NuMA also binds LGN, which acts as a bridge between the spindle and the cellular cortex through its interaction with plasma membrane-associated Gαi (di Pietro *et al.*, 2016). Spindle regulators such as LGN and NuMA are necessary to properly orient cell divisions in the epidermis during embryonic development, and loss of these components impairs epidermal stratification (Lechler and Fuchs, 2005; Williams *et al.*, 2011, 2014). The functions of these components are highly context-dependent. For example, LGN loss in the back-skin epidermis and most oral epithelia greatly reduces perpendicular divisions, and leads to delayed stratification (Williams *et al.*, 2011, 2014; Byrd *et al.*, 2016), whereas in the dorsal tongue papillary placodes, it causes a marked increase in perpendicular divisions at the expense of parallel divisions, which disrupts papillary morphogenesis (Byrd *et al.*, 2016). Finally, in developing hair follicles, loss of LGN has only minor effects on division orientation (Byrd *et al.*, 2016).

In contrast to developing tissues, few studies have addressed how components of the spindle orientation machinery contribute to adult tissue homeostasis or influence tumorigenesis. In adult back skin, ear, and hindpaw epithelia, basal cells divide mostly parallel to the basement membrane, with varying proportions of oblique and perpendicular divisions depending on the tissue (Ipponjima *et al.*, 2016). In thicker tail epidermis however, adult basal cells often undergo oblique divisions, highlighting the context-dependence of oriented cell division (Ipponjima *et al.*, 2016). It is not yet clear how components of the spindle orientation machinery regulate division angles at any of these body sites.

Key features of tumorigenesis include loss of cell polarity and disruption of tissue organization, both of which are intrinsically linked to spindle orientation. Polarity complexes, as well as environmental cues, can regulate spindle orientation. In turn, mitotic spindle orientation, by determining daughter cell positioning, can impact tissue organization (Lechler and Mapelli, 2021). In addition, spindle orientation defects have been associated with oncogenic mutations in mammalian cells (Fleming *et al.*, 2009; Thoma *et al.*, 2009; Quyn *et al.*, 2010; Hehnly *et al.*, 2015). These associations suggest a potential role of spindle orientation in tumorigenesis but whether spindle orientation defects are a cause or a consequence of tumorigenesis remains to be determined (Seldin and Macara, 2017). Emerging evidence suggests that spindle regulators can indeed impact epidermal tumor development. A recent study combining functional loss of NuMA with constitutively active K-Ras revealed that spindle orientation plays a crucial role in managing oncogenic mutations in the epidermis by compensating for decreased rates of differentiation (Morrow *et al.*, 2019). The role of LGN, however, has not been studied in adult epidermis, either in a normal homeostatic context or in conjunction with oncogenic perturbations.

The PI3K-AKT axis is one of the most frequently altered pathways in cancer (Chalhoub and Baker, 2009). PTEN is a lipid phosphatase that acts as a major negative regulator of the PI3K pathway and is a frequently inactivated tumor suppressor in cancer. Germline PTEN mutations have been linked to a variety of syndromes, grouped under the term PTEN Hamartoma Tumor Syndrome, characterized by benign growths and increased risk of developing certain types of cancers (Hollander *et al.*, 2011). Global loss of PTEN in the epidermis results in epidermal hyperplasia and tumor formation (Suzuki *et al.*, 2003). This may be driven at least in part by accelerated proliferation and differentiation behaviors that occur as a consequence of PI3K activation pathway in basal cells (Ying *et al.*, 2018). There have been limited reports on the role of spindle orientation regulators in the context of oncogenic mutations. Given the role of PTEN loss in the regulation of basal differentiation in tumorigenesis, we set out to determine whether disrupted spindle orientation could lead to a worsened prognosis in a PTEN-deficient context.

Here, we examine the role of LGN both during adult skin homeostasis as well as in the context of oncogenic perturbation, by crossing LGN –/– mice with a PTEN-deficient mouse model. We show that loss of LGN in PTEN-deficient conditions accelerates tumor growth on the snout and eyelids. In contrast, loss of LGN in otherwise wild-type adult skin disrupts spindle orientation without affecting tissue architecture. By evaluating multiple timepoints to characterize the PTEN-deficient epidermis, we find that loss of LGN randomizes spindle orientation. In addition, a subset of LGN- and PTEN-deficient mice have an increased amount of proliferative spinous cells, which may be involved in tumorigenesis.

## RESULTS AND DISCUSSION

### LGN loss accelerates epidermal tumor initiation after PTEN deletion

**FIGURE 1:**
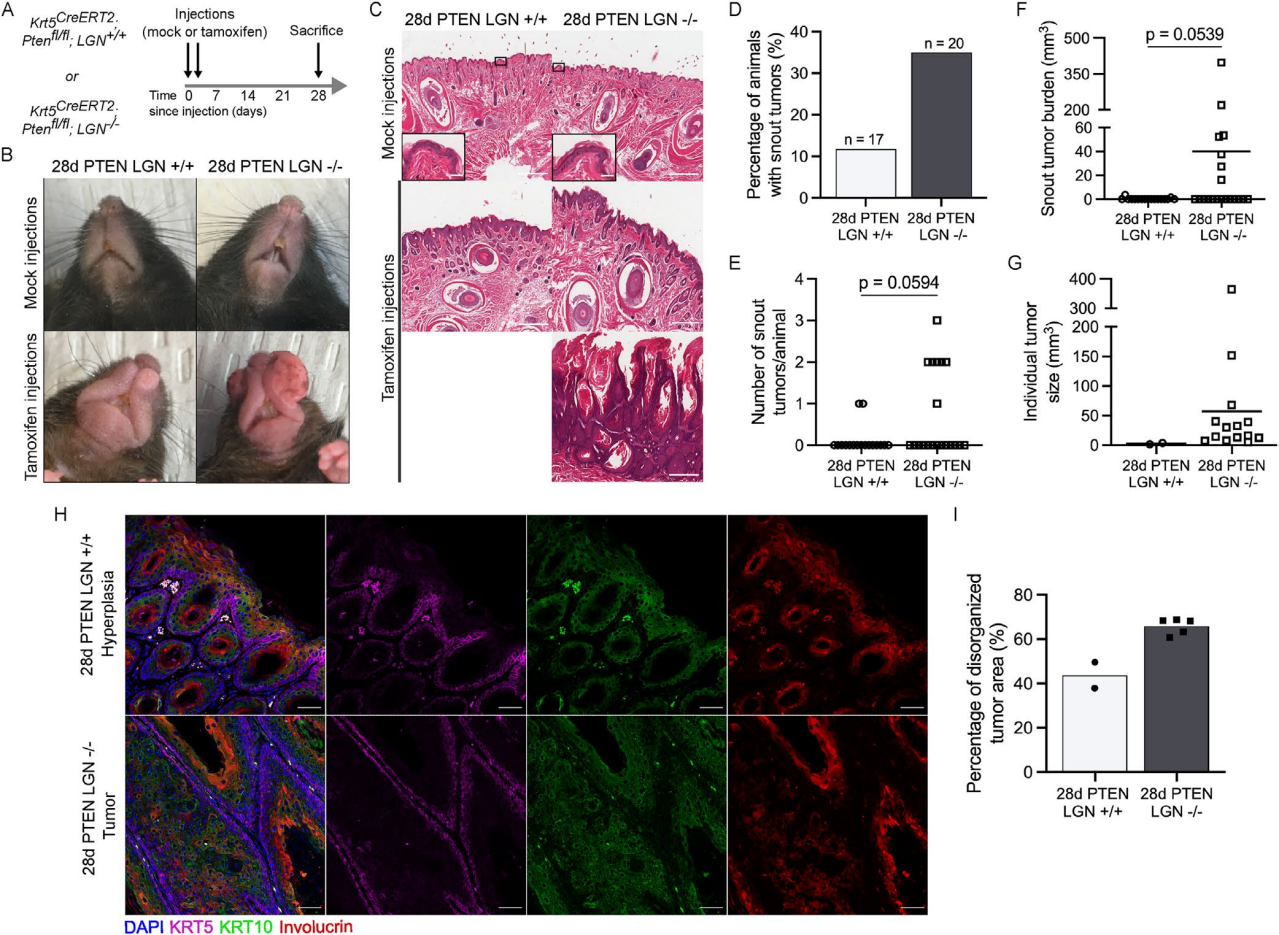
Loss of LGN accelerates tumorigenesis in K5CreERT2; PTENfl/fl mouse epidermis. (A) Krt5CreERT2; PTENfl/fl; LGN +/+ and Krt5CreERT2; PTENfl/fl; LGN –/– mice were injected with corn oil (mock injections) or injected with tamoxifen and analysed after 28 days. (B and C) Representative images of the snouts (B) and H&E staining of lip epidermis (C) are shown. H&E images magnified 5×, scale bar = 200 μm, insets magnified 20×, scale bar = 50 μm. (D–G) Quantifications of the proportion of tamoxifen-injected mice with tumors on their snouts (D), number of snout tumors per mouse (E), total tumor volume on the snout (F) and individual tumor size (G) in mm^3^. *N* = 17 mice for 28d PTEN LGN +/+ and *N* = 20 mice for 28d PTEN LGN –/–, tested with two-tailed Mann-Whitney test. (H) Maximum intensity projection of immunostaining of hyperplastic epithelium (28d PTEN LGN +/+ mouse, i) and tumor tissue (28d PTEN LGN –/– mouse, ii) using lineage markers KRT5, KRT10 and Involucrin. Scale bar = 50 μm. (I) Percentage of area displaying cellular disorganization in tumor tissue of 28d PTEN LGN +/+ and 28d PTEN LGN –/– mice. *N* = 2 tumors for 28d PTEN LGN +/+, *n* = 5 tumors for 28d PTEN LGN –/–.

To investigate the relationship between spindle orientation and tumor development in the skin, we induced PTEN deletion in the adult epidermis of *Krt5^CreERT2^*; *Pten^fl/fl^* mice with either an LGN^+/+^ or LGN^–/–^ background (Figure 1A). We confirmed PTEN deletion by genotyping FACS-sorted basal cells of the footpad epidermis (Supplemental Figure S1A). Four weeks after tamoxifen-induced Cre recombinase activation, 12% of PTEN LGN +/+ mice had developed tumors on their lips, while 35% of the PTEN LGN –/– mice had developed multiple large tumors on their snout (lips, chin, and mystacial pads; Figure 1, A–D). Tumor-bearing PTEN LGN –/– mice developed an average of two, and up to three, tumors on their snout, while PTEN LGN +/+ mice presented with a single snout tumor at most (Figure 1E). In addition, the snout tumor burden for PTEN LGN +/+ mice was over 10-fold smaller than for the PTEN LGN –/– mice, with average volumes of 0.3 mm^3^ and 40.2 mm^3^, respectively (Figure 1F). This was most striking in the largest tumor of each genotype, with a 110-fold difference in size between the PTEN LGN +/+ group (3.6 mm^3^) and the PTEN LGN –/– animals (397.6 mm^3^; Figure 1G). In addition to the snout, both groups developed tumors on their eyelids (Supplemental Figure S1B). While 27% of PTEN LGN +/+ mice developed an eyelid tumor, that percentage was 79% in PTEN LGN –/– mice. Further, the PTEN LGN +/+ mice had at most one eyelid affected whereas both eyelids were affected in 26% of PTEN LGN –/– mice (Supplemental Figure S1C). The eyelid tumor size was similar in 28d PTEN LGN +/+ and 28d PTEN LGN –/– (Supplemental Figure S1D). Interestingly, all animals had developed tumors on their paws, regardless of their genotype (Supplemental Figure S1E).

Histological examination of snout (lips and mystacial pads) tissue by H&E revealed a hyperplastic epidermis in PTEN-deficient tissue regardless of LGN background (Figure 1C), which is consistent with reports of hyperplasia in other regions of the skin when *Pten* is inactivated (Suzuki *et al.*, 2003). Examination of H&E sections from PTEN LGN –/– tumors revealed that they were benign papillomas. We performed an immunofluorescence assay on tissue sections to further characterize tissue organization within the tumors, using Keratin 5 (KRT5) to mark basal cells, Keratin 10 (KRT10) to mark spinous cells, and Involucrin to mark granular cells. The tumor tissue was found to have a mixed tissue architecture phenotype, with some regions organized into clearly stratified epithelial structures, and others with loose, mixed cell types without identifiable stratification (Figure 1H). We found that for all tumors, at least 50% of the tissue showed some signs of cellular disorganization, which we defined as the presence of differentiation markers in the inappropriate epidermal layer (e.g., involucrin+ cells in spinous layer). In PTEN LGN –/– tumors, 60–70% of the tumor tissue displayed some level of cellular disorganization (Figure 1G). Although the small number of tumors that formed in PTEN LGN +/+ mice limited our ability to perform statistical comparisons, visual inspection indicated that PTEN LGN –/– and PTEN LGN +/+ tumors had similar amounts of cellular disorganization. These results show that the absence of LGN, combined with PTEN loss, markedly accelerates tumor onset in distinct anatomical skin regions.

### Loss of LGN decreases planar divisions in adult snout epidermis

**FIGURE 2:**
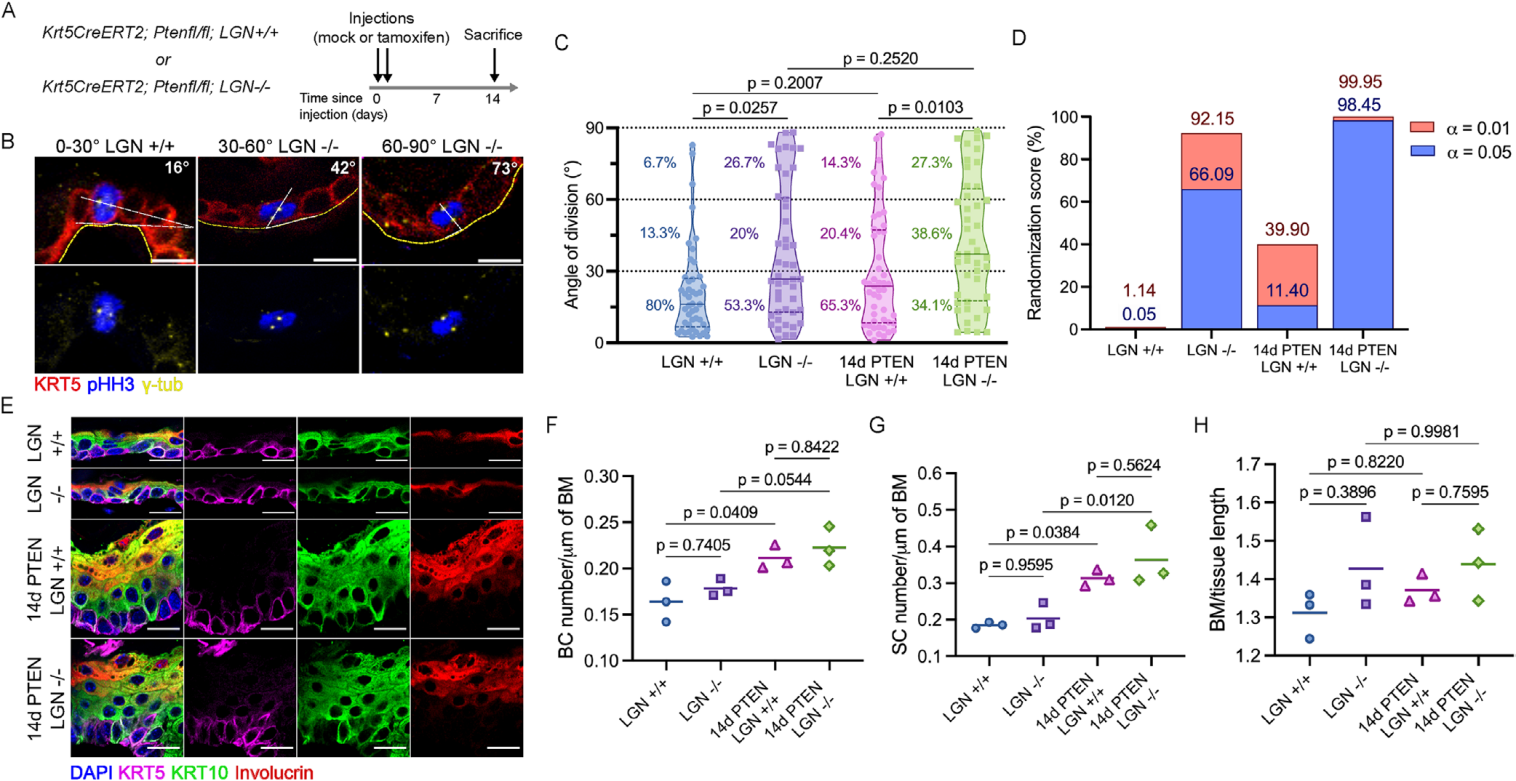
Loss of LGN in adult snout epidermis alters spindle orientation without affecting tissue architecture. (A) Krt5CreERT2; PTENfl/fl; LGN +/+ and Krt5CreERT2; PTENfl/fl; LGN –/– mice were injected with corn oil (mock injections) or injected with tamoxifen and analysed after 14 days. (B) Representative immunostaining of dividing cells from the three angle bins used for quantifications with basal (KRT5), mitotic (pHH3), and spindle pole (γ-tubulin) markers. The yellow dashed line outlines the basal lamina and the white dashed line traces the angle of division. Scale bar = 10 μm. (C) Distribution of the mitotic spindle angles with percentage of angles indicated for the 0–30°, 30–60°, and 60–90° bins. Solid line represents the median, dashed lines represent the 25th and 75th percentiles. *N* = 45 divisions from three mice each for LGN +/+ and LGN –/–, *n* = 49 divisions from four mice for 14d PTEN LGN +/+ and *n* = 44 from four mice for 14d PTEN LGN –/–, each pair was tested using Kolmogorov-Smirnov test. (D) Bar graph representing the randomization score of the distributions. The randomization score is calculated by comparing each experimental distribution to 100,000 random distributions of the same size (*n* = 45, random numbers between 0 and 90), and performing the Kolmogorov-Smirnov test. The randomization score is the percentage of these *p* values that are larger than α, which measures if the experimental distribution is statistically equivalent to a random distribution. (E) Representative images of the snout epidermis from PTEN LGN +/+ and PTEN LGN –/– mice 14 days after mock or tamoxifen injections, stained with DAPI and lineage markers KRT5, KRT10, and Involucrin. Scale bar = 20 μm. (F–H) Quantification of the basal cell layer density (F), spinous cell layer density (G) and tissue folding (H). Cell density is assessed by the number of basal (KRT5+) or spinous (KRT10+) cells per μm of basement membrane. Tissue folding is the ratio of the basement membrane length over total tissue. >300 cells/animal, three mice/condition, tested with one-way ANOVA.

Our results demonstrate that LGN deficiency in the context of PTEN loss accelerates benign tumor initiation. To further understand the consequences of LGN deficiency in one of the epidermal regions prone to accelerated tumor formation, we focused on snout epidermis. To understand tissue-wide and cellular changes that lead to tumor growth when PTEN and LGN are both lost, we evaluated tissue architecture at different stages in PTEN-deficient animals before tumors were visible. PTEN LGN +/+ and PTEN LGN –/– snout tissues were collected at 7 days (7d) and 14 days (14d) post-tamoxifen injections and immunostained for phosphorylated AKT (pAKT), a downstream target of PTEN, as well as KRT5. At 7d post-injections, there were no pAKT+ cells and the epidermal tissue architecture was similar to the mock-injected control samples. However, at 14d post-injections the epithelium was markedly thicker and pAKT was expressed throughout the suprabasal layer as well as in a subset of basal cells. (Supplemental Figure S2). We therefore chose the 14d timepoint for further characterization (Figure 2A)*.*

LGN is one of the key regulators of spindle orientation. We therefore sought to establish the effects of LGN loss on mitotic basal cells in PTEN wild type and PTEN-deficient snout epidermis (lips and mystacial pads) by measuring angles of division. We used γ-tubulin to identify the spindle poles of mitotic (phospho-Histone H3, pHH3+) cells and traced the angle of spindle orientation using the basal layer (KRT5+) as a reference. We classified divisions as parallel to the basement membrane (≤ 30°), perpendicular to the basement membrane (≥ 60°), or oblique (30 to 60°; Figure 2B). We found that in wild type tissue, 80% of cell divisions were parallel to the basement membrane, similar to adult epidermis from the dorsal skin, ear, and paw (Ipponjima *et al.*, 2016; Morrow *et al.*, 2019). However, loss of LGN dramatically decreased the proportion of parallel divisions from 80 to 53%, and sharply increased the proportion of perpendicular divisions from 7 to 27%. It also modestly increased oblique divisions (from 13 to 20%; Figure 2, B and C). We then measured the angle of spindle orientation in PTEN mutant tissue to determine whether loss of PTEN together with LGN deletion further alters spindle orientation. While 14d PTEN LGN +/+ basal cells continue to divide predominantly parallel to the basement membrane (65% under 30°), there is a small increase in oblique (20%) and perpendicular (14%) divisions, compared with the wild type condition. In 14d PTEN LGN –/– tissue however, the skew towards parallel divisions completely disappears and the angle distribution seems fully randomized (parallel: 34%, oblique: 38%, perpendicular: 27%; Figure 2C).

The change in distribution of cell division angles led us to wonder whether loss of LGN leads to randomization of the angle of spindle orientation. To determine whether the distribution of mitotic spindle orientation in LGN –/– mice was randomized, we compiled randomization scores for each distribution (see *Methods*). The randomization score represents the probability that the experimental distribution is statistically equivalent to a random distribution; the higher the score, the more randomized the distribution. We computed the randomization score using a low or high significance threshold (low: α = 0.05; high: α = 0.01). The low significance threshold has a higher stringency for randomness than the high significance threshold does. As expected, this analysis confirmed that the LGN +/+ distribution is not randomized, with a characteristic strong skew towards horizontal divisions. The consistency between the randomization scores with α = 0.05 and α = 0.01 shows that this result is robust. The LGN –/– distribution is substantially more randomized than the LGN +/+, in line with the significant increase in vertical and oblique divisions observed in that condition (Figure 2D). This analysis shows that the loss of LGN partially randomizes spindle orientation as a moderate preference for parallel divisions was retained. Further, the randomization scores indicate that the 14d PTEN LGN +/+ distribution has low levels of randomization. In 14d PTEN LGN –/– tissue however, the randomization scores of around 99% confirm that the angle distribution is fully randomized (Figure 2D). Unlike previous studies of embryonic epidermis where LGN facilitates perpendicular divisions (Williams *et al.*, 2011; Byrd *et al.*, 2016), our results demonstrate that LGN in the adult snout epidermis contributes to the maintenance of parallel divisions. In addition, this analysis shows that while PTEN loss does not significantly impact spindle orientation, the combined loss of PTEN and LGN randomizes spindle orientation in basal cells.

We next sought to assess how these substantial changes in spindle orientation impact tissue architecture in LGN mutants. To visualize tissue architecture, we performed immunostaining on tissue sections with KRT5, KRT10, and Involucrin and quantified the basal and spinous cellular densities (Figure 2E). Surprisingly, neither basal nor spinous compartments were affected by LGN loss in wild type PTEN conditions; both densities were similar to the wild type control (basal: LGN +/+ 0.16 vs. LGN –/– 0.18 cells per μm of basement membrane, spinous: LGN +/+ 0.19 vs. LGN –/– 0.20 cells per μm of basement membrane). Upon PTEN loss, the basal layer density increased by 31% in LGN +/+ tissue and 29% in LGN –/– tissue, while the spinous layer underwent a considerable expansion with a density increase of 72% in LGN +/+ tissue and 80% in LGN –/–tissue. Quantification of the basal and spinous layer densities in 14d PTEN LGN +/+ and 14d PTEN LGN –/– tissue (basal: 0.21 vs. 0.22 cells/μm of basement membrane, respectively; spinous: 0.31 vs. 0.36, respectively) did not reveal LGN-dependent differences (Figure 2, F and G). While cellular density captures differences in cell packing, it does not account for the presence of “extra” tissue. To test for this, we measured tissue folding and found that the levels of folding are similar in both the LGN +/+ and LGN –/– tissue, and the 14d PTEN LGN +/+ and 14d PTEN LGN –/–, excluding a difference in the absolute number of cells (Figure 2H). Together, these results indicate that although LGN deficiency substantially affects spindle orientation in homeostatic and PTEN-deficient epidermis, it does not impact tissue architecture up to the 14d stage.

### Cells positioned suprabasally following oblique divisions can reintegrate into the basal layer

**FIGURE 3:**
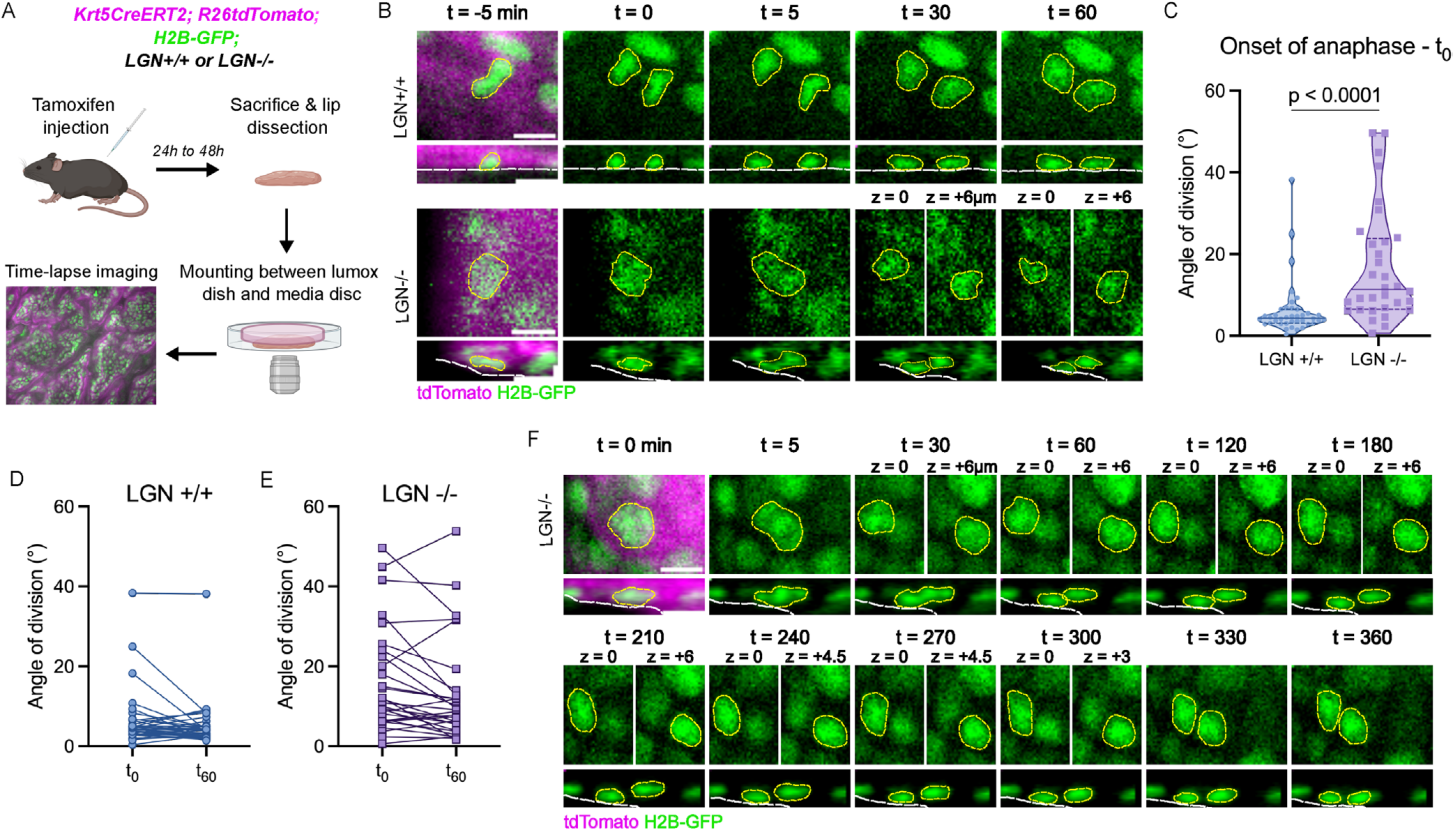
Cells positioned suprabasally following oblique divisions can reintegrate into the basal layer in snout epidermis. (A) Krt5CreERT2; R26tdTomato; H2B-GFP; LGN+/+ or LGN–/– mice were injected with tamoxifen 24 to 48 h before sacrifice. The outer lips were dissected, mounted between a media disk and a lumox membrane, and imaged with a spinning disk confocal microscope. (B) Time-lapse images of a planar division (top) and oblique division (bottom), shown in the top-down view (upper images) and reslice view (lower images). Dividing cell and daughter cells are outlined in yellow, the basement membrane is outlined in white. Scale bar = 10 μm. (C) Distribution of the spindle angles at onset of anaphase (first frame of anaphase) measured from reslice views in live-imaging movies. Solid line represents the median, dashed lines represent the 25th and 75th percentiles. *N* = 39 divisions from two mice for LGN +/+, *n* = 32 from two mice for LGN –/–, tested with Kolmogorov-Smirnov test. (D) Division angles in LGN +/+ tissue measured at onset of anaphase (t0) and 1 h later (t60). *N* = 36 from two mice. (E) Division angles in LGN –/– tissue measured at onset of anaphase (t0) and 1 h later (t60). *N* = 29 from two mice. (F) Time-lapse images of an oblique division leading to a suprabasal daughter that moves down towards the basal layer over several hours following the division, shown in the top-down view (upper images) and reslice view (lower images). Dividing cell and daughter cells are outlined in yellow, the basement membrane is outlined in white. Scale bar = 10 μm.

Our data show that in LGN-deficient tissue, tissue architecture is maintained despite major changes in mitotic spindle orientation. We wondered whether non-planar divisions give rise to daughter cells that are retained in the spinous layer, or whether the position of daughter cells can resolve to become basal. To track divisions and visualize their outcome, we optimized a protocol for live-imaging of adult skin explants by adapting the protocols described in Cetera *et al.* (2018) and Lough *et al.* (2019). We used *Krt5^CreERT2^; R26^tdTomato^; H2B-GFP; LGN* +/+ or *LGN* –/– mice in which chromosomes are labeled with GFP, and tdTomato expression was used to mark the epidermis and identify cells in the basal layer. We were able to image lip explants every 5 min for 13–15 h, identify dividing basal cells, and visualize mitoses (Figure 3, A and B). We observed basal cells dividing even at late stages of the time-lapse, confirming that the tissue was healthy for the duration of the experiment.

We first compared the division angles observed during live-imaging to our earlier results in fixed tissue by measuring the angle of division at the onset of anaphase. We found that in both LGN wild type and mutant tissue, the distribution of angles was narrower than in measurements made in tissue sections; no angles above 50 degrees were recorded (Figure 3C). This could reflect changes in spindle orientation between metaphase (tissue section) and anaphase (live-imaging), as the spindle firmly anchors in anaphase. Improved accuracy of angle measurement with live-imaging could also contribute to this difference. Similar to the measurements in tissue sections however, almost all divisions in WT tissue are planar (95% of divisions under 20°) whereas loss of LGN decreases the proportion of planar divisions and increases the proportion of oblique divisions (66% under 20°; Figure 3, B and C).

In wild type embryonic epidermis, basal divisions can start out with an oblique orientation at metaphase and anaphase. By the end of telophase however, the division is corrected to a planar or perpendicular orientation (Lough *et al.*, 2019). To assess whether a similar telophase correction process could explain the normal tissue architecture in LGN mutants, we measured the angle of division 1 h after the onset of anaphase (Figure 3, D and E). We found that most oblique divisions do not resolve within 1 h of anaphase, which indicates that telophase correction is not the primary mechanism of rectifying these oblique divisions. To determine whether the daughter cells generated by these oblique divisions remained in their positions, we tracked them over several hours post-anaphase when possible. We found that some of the suprabasally-positioned cells move downwards and realign with the basal daughter hours after the end of mitosis (Figure 3F). We did not observe this behaviour in all cells (two out of five suprabasal LGN –/– cells tracked for more than 6 h), which could be due to limits on the duration of our imaging window or because this does not happen in all cells. Reintegration of mispositioned cells into epithelia, whether during normal development or following spindle orientation defects, happens during invertebrate and mammalian development (Bergstralh *et al.*, 2015; Pfannenstein and Macara, 2023). Our data indicates that this also happens in mammalian adult tissue. This capacity of daughter cells to move back to the basal layer provides a potential mechanism that accounts for the lack of tissue architecture defects in LGN mutants.

### Homeostatic processes are not affected by LGN loss in basal cells of adult-snout epidermis

**FIGURE 4:**
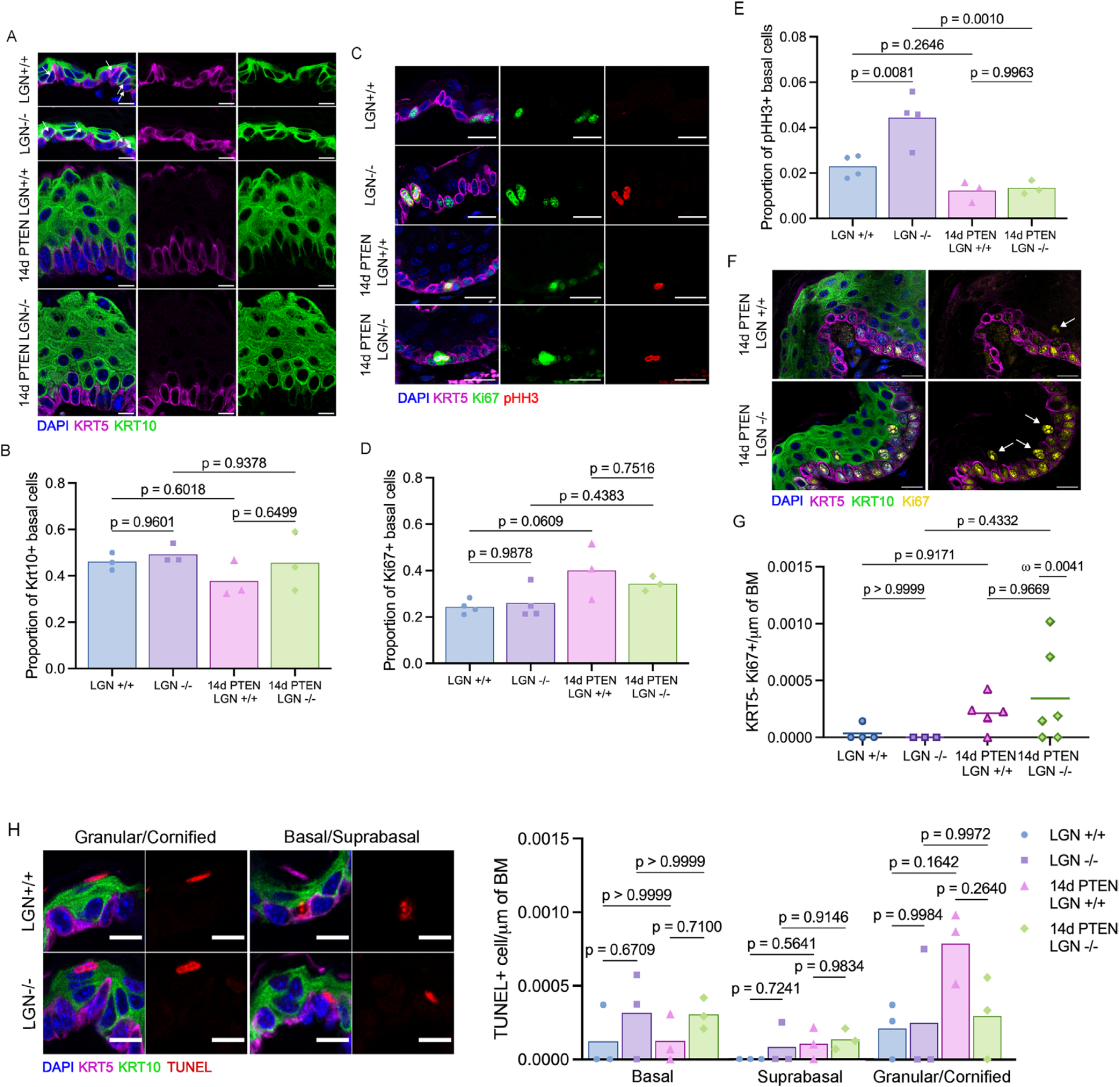
Homeostatic processes are not affected by LGN loss in basal cells of adult snout epidermis. (A) Representative images of the snout epidermis from PTEN LGN +/+ and PTEN LGN –/– mice 14 days after mock or tamoxifen injections, stained with DAPI and lineage markers KRT5 and KRT10. Arrows mark examples of cells expressing both KRT5 and KRT10. Scale bar = 10 μm. (B) Differentiation rate assessed by the proportion of KRT10+ basal cells. >300 cells/animal, three mice/condition, tested with one-way ANOVA. (C) Representative images of the epidermis stained with DAPI, KRT5, Ki67, and pHH3. Scale bar = 20 μm. (D and E) Proportion of proliferative (Ki67+) basal cells (D) and mitotic (pHH3+) basal cells (E). For LGN +/+ and LGN –/–, > 600 cells/animal, four mice/condition; for 14d PTEN LGN +/+ and 14d PTEN LGN –/–, >1400 cells/animal, three mice/condition; tested with one-way ANOVA. (F) Representative images of the epidermis stained with DAPI, KRT5, KRT10, and Ki67. White arrows show Ki67+ KRT10+ KRT5- cells. Scale bar = 20 μm. (G) Frequency of proliferative spinous cells in the tissue. >3500 μm/animal; tested with one-way ANOVA. Omega is the result of an outlier test; it is the likelihood to obtain values above 0.007 in a Gaussian distribution with the mean and SD of the 14d PTEN LGN +/+ condition. (H) Representative images of TUNEL+ cells in the epidermis of LGN +/+ and LGN –/– mice stained with lineage markers KRT5 and KRT10. Scale bar = 10 μm. (I) Frequency of TUNEL+ cells in basal, suprasabal, and granular/cornified layers. >2500 μm/animal; tested with one-way ANOVA for each layer.

Our live-imaging analysis demonstrated that mispositioned cells can move back to the basal layer in wild type PTEN conditions. We wondered whether other processes may compensate for the changes in spindle orientation within the tissue, especially when PTEN is lost. Proliferation (basal cell gain) and differentiation/delamination (basal cell loss) are the primary mechanisms that regulate the basal homeostasis in the adult epidermis. Apoptosis (cell loss), although rare, could also contribute to managing spindle orientation defects within the tissue. To determine the frequency of basal cells initiating differentiation, we counted the proportion of basal cells expressing the spinous marker KRT10. The proportion of KRT10+ basal cells was similar in LGN +/+ (46%) and LGN –/– (49%) epidermis, indicating that loss of LGN does not impact differentiation in snout epidermis. Similarly, the frequency of basal cells initiating differentiation was comparable between the 14d PTEN LGN +/+ (38%) and 14d PTEN LGN –/– (46%) epidermis (Figure 4, A and B). Interestingly, despite recent evidence that activation of the PI3K-AKT pathway can promote differentiation (Ying *et al.*, 2018), loss of PTEN did not impact the proportion of basal cells undergoing differentiation at this stage.

To determine the rate of proliferation in the basal layer, we stained with Ki67, a general proliferation marker, and pHH3, a mitotic marker (Figure 4C). The proportion of Ki67+ cells was similar in both PTEN wild type conditions (25 vs. 26% for LGN +/+ and –/–, respectively). Loss of PTEN led to an increase in Ki67+ basal cells, but the proportion of Ki67+ cells in the 14d PTEN LGN +/+ tissue (40%) was similar to what we observed in the 14d PTEN LGN –/– tissue (34%; Figure 4D). Surprisingly, we found that while 2% of basal cells were pHH3+ in the LGN +/+ tissue, that number doubled to 4% in the LGN –/– epidermis (Figure 4E). This difference could be explained by an increase in proliferation too small to be picked up by Ki67, or by a lengthening of the mitotic phase caused by loss of LGN. LGN has been found to affect the length of mitosis in vitro and could have a similar effect in vivo (Kaushik *et al.*, 2003). This suggests that LGN loss affects mitosis in the epidermis, either by increasing proliferation or by affecting mitosis kinetics. Surprisingly, the proportion of pHH3+ cells in 14d PTEN LGN +/+ and 14d PTEN LGN –/– tissue was similar (1.6 and 1.3%, respectively), and not significantly different from the LGN +/+ tissue (Figure 4E). PTEN is involved in the regulation of mitotic and spindle assembly checkpoints, and PTEN loss in vitro accelerates mitosis (Choi *et al.*, 2017; Liu *et al.*, 2017). This could explain why the proportion of pHH3+ basal cells are not higher in the PTEN conditions. Altogether, our analysis of basal cell behaviors indicates that loss of LGN does not affect either proliferation or differentiation in PTEN-deficient tissue.

We further examined the 14d PTEN epidermis for abnormal changes and found rare suprabasal Ki67+ spinous (Krt5-) cells (Figure 4F). We quantified the frequency of these cells and found that it is elevated up to fivefold in a third of 14d PTEN LGN –/– animals, compared with the 14d PTEN LGN +/+ tissue (Figure 4G). This corresponds to the phenotype at 28d, where a third of the PTEN LGN –/– mice have developed tumors (Figure 1D). To investigate these spinous proliferative cells at later stages, we quantified their frequency in 28d PTEN tumors and found that they are more common than in 14d tissue (Supplemental Figure S3).

We next wondered whether daughter cells from abnormal divisions could be eliminated through apoptosis. To assess cell death in the tissue, we performed a TUNEL assay and observed sparse TUNEL+ cells at the uppermost differentiated cell layer, which is consistent with cornification (Demerjian *et al.*, 2008) and rare TUNEL+ cells in the basal and suprabasal layers, consistent with apoptotic cells (Figure 4H). We quantified cell death in the basal, suprabasal, and granular/cornified layers. TUNEL+ cells were rare, and no significant differences were observed between the four conditions (Figure 4I).

These results indicate that loss of LGN and the concomitant increase in perpendicular and oblique divisions does not affect differentiation or apoptosis, but does affect proliferation. Further, loss of LGN in PTEN-deficient epidermis randomizes spindle orientation but does not affect other homeostatic processes in the basal layer at 14d. It is, however, associated with an increase in proliferative spinous cells in a subset of mice.

Here, we demonstrate that loss of LGN accelerates tumor initiation on the snout in a PTEN-deficient mouse model. This supports a model in which randomization of cell division orientation promotes tumor onset, potentially in part by generating proliferative spinous cells. Morrow *et al.* (2019) reported that combining loss of NuMA function with oncogenic K-Ras in the footpad epithelium produced a similar effect. However, the mechanistic changes that lead to increased tumorigenesis differ. In that study, expression of oncogenic K-Ras increases perpendicular divisions in the basal layer of the footpad in a NuMA-dependent manner. Loss of regulated spindle orientation by disruption of NuMA functions prevents vertical orientation of the spindle, leading to expansion of the basal layer and tissue overgrowth. In contrast, in snout epidermis, spindle orientation is not significantly impacted by the loss of PTEN. Moreover, loss of LGN randomizes spindle orientation, which increases oblique and perpendicular divisions. This shift in orientation of division may result in local accumulation of proliferative spinous daughter cells from those nonplanar divisions. We hypothesize that these rare suprabasal proliferation events may further disturb epithelial homeostasis and accelerate tumor formation.

Notably, the role of LGN in adult skin appears to differ from its established role in promoting perpendicular divisions during the initial development and stratification of the epidermis. This may be explained by the differing needs of the developing epidermis and the adult homeostatic tissue. While perpendicular divisions are needed for stratification in the embryo, adult basal cells of the epidermis divide mostly in plane. It is likely that the core machinery involved in regulating the orientation of the spindle during development is also involved in supporting planar divisions in adult tissue. We propose that LGN localizes to the basolateral cortex, and through its canonical interactions with NuMA and Gαi, participates in the anchoring of the spindle poles to promote planar divisions. Basolateral localization of LGN could be achieved several ways. In embryonic skin, Inscuteable (Insc) has been shown to recruit LGN apically to promote perpendicular divisions (Williams *et al.*, 2014). In adult tissue however, single-cell RNA-sequencing evidence suggests that, unlike LGN, Insc is not expressed in proliferating basal cells (Joost *et al.*, 2020). In the absence of Insc, there may not be a way for LGN to be recruited to the apical domain. Conversely, SAPCD2, which has been shown to exclude LGN from the apical domain in mouse retinal progenitor cells (Chiu *et al.*, 2016), is expressed in dividing basal cells of the adult epidermis (Joost *et al.*, 2020). If SAPCD2 carries out a similar function in epidermal cells, it provides a mechanism for ensuring LGN localization at the basolateral cortex.

Interestingly, there is evidence that PTEN can significantly alter spindle orientation in nonpolarized, cultured HeLa cells (Toyoshima *et al.*, 2007). In that system, spindle orientation is controlled by integrin-dependent cell-substrate adhesion. Toyoshima et al propose that integrin-mediated midcortical accumulation of PtdIns(3,4,5)P3, a PTEN substrate, is necessary for recruitment of dynactin at the midcortex and planar spindle orientation. Our results suggest that spindle orientation in adult epidermis is primarily regulated by alternate mechanisms. Multiple studies shows that the NuMA-LGN-Gαi complex, regulated by polarity proteins, controls spindle orientation in embryonic skin (Lechler and Fuchs, 2005; Williams *et al.*, 2011, 2014), which provides an explanation for the negligible effect of PTEN loss on spindle orientation.

Constitutive clonal oncogenic activation of PI3K signalling has been shown to promote differentiation in epidermal basal cells (Ying *et al.*, 2018). In our model, loss of PTEN (PI3K activation) leads to tumor growth on the paws, snout, and eyelids. In snout tissue, PTEN loss leads to a rapid expansion of all cell layers in the epidermis between day 7 and day 14 post-injections, suggesting that loss of PTEN initially causes an increase in differentiation. This increase is temporary however, as the differentiation rate is back to PTEN WT rates by day 14, and the epidermis does not markedly thicken further between day 14 and day 28, with the exception of regions where tumors have formed. This suggests that after this initial burst of differentiation, the hyperplastic epidermis reaches a temporary homeostatic state. Randomization of spindle orientation may then disturb this transitory equilibrium by increasing the amount of proliferative spinous cells and disrupting tissue organization, contributing to tumor initiation. A third of PTEN LGN –/– animals bear tumors on their snout at 28d. For tumors to be present at 28d, the same proportion of animals should exhibit changes linked to tumor initiation at earlier timepoints. We indeed observe that a third of PTEN LGN –/– mice have higher amounts of proliferative spinous cells in the snout epidermis at 14d. We further confirmed that these proliferative spinous cells are associated with tumors, by showing that they are common in tumor tissue at 28d. This supports a model where randomization of spindle orientation may generate spinous proliferative cells that could contribute to tumor initiation. However, further work would be needed to confirm this model. Emerging data show that up to a third of normal epidermal cells in aged human skin harbor oncogenic somatic mutations, despite the tissue being phenotypically normal (Martincorena *et al.*, 2015). This suggests that other mutations or defects are necessary to initiate tumorigenesis. Our results suggest that spindle orientation defects may facilitate tumor initiation by collaborating with oncogenic mutations.

## MATERIALS AND METHODS

Request a protocol through *Bio-protocol*.

### Mice

Experimental mice were maintained in a C57BL/6 background mixed with C3H. The *Krt5^CreERT2^* (Van Keymeulen *et al.*, 2011), *Pten^fl^* (Trotman *et al.*, 2003), LGN^–/–^ (Tarchini *et al.*, 2013), *R26^tdTomato^* (Madisen *et al.*, 2010), and H2B-GFP (Hadjantonakis and Papaioannou, 2004) mice were described previously.

All experiments were performed with 8-wk to 14-wk-old males, except live-imaging experiments, where 1 LGN^–/–^ female was used.

Mice were given two intraperitoneal injections, 48 h apart, of either corn oil (mock-injection) or 3 mg of tamoxifen (Toronto Research Chemicals, #T006000) dissolved in corn oil for Cre recombinase activation. Mock-injected *Krt5^CreERT2^*; *Pten^fl/fl^*; LGN^+/+^, or LGN^–/–^ animals are referred to as LGN^+/+^ or LGN^–/–^ mice.

All animal procedures were approved by McGill University Animal Care Committee according to the Canadian Council on Animal Care guidelines for use of laboratory animals in biological research.

Tumor size was measured from pictures taken of the mice from different angles. The pictures allowed to measure the volume of snout tumors and the area of eyelid tumors (the size and location of these tumors made volume measurements impossible).

### FACS and genotyping

Animals were sacrificed at 21 days post-injections. The skin of the footpad was dissected out and floated, dermis side down, on 5 mg/ml dispase at 37°C for 20 min. The epidermis was peeled from the dermis and minced with scissors in 0.25% trypsin+EDTA solution for 2 min. It was then further incubated in the solution for 5 min at 37°C. The trypsin was neutralized with a DMEM+10% fetal bovine serum (FBS) solution and cells were filtered first through a 100 µm strainer into a phosphate-buffered saline (PBS)+0.5%FBS solution and again through a 70 µm strainer. After spinning at 200 rcf for 5 min, the pellet was resuspended in PBS+3% FBS+2 mM EDTA. For staining, the single cell suspension was incubated with anti-Cd49f (1:200, Invitrogen, #11-0495-82) and Fixable Viability Dye eFluor 780 (1:1000, Invitrogen, #65-0865-14) at 4°C for 30 min. Live Cd49f^+^ cells were sorted with a BD FACSAria Fusion flow cytometer (BD Biosciences). DNA extraction was performed using the AllPrep DNA/RNA Micro Kit (Qiagen, #80284), following manufacturer’s protocol. For genotyping, PCR was performed with the following primers. For the floxed allele, forward: CATCA­TCCTTGCTCTCAGTGT; reverse: GCACTGGGTAGCAAGATCAC. For the deleted allele, forward: AAAAGTTCCCCTGCTGATGA­TTTGT; reverse: CCCCCAAGTCAATTGTTAGGTCTGT.

### Tissue processing and immunofluorescence

Samples were fixed for 48 h in 4% PFA, embedded in paraffin and 4–8 um sections were generated.

For immunofluorescence, slides were warmed up to 50°C and immersed in HistoClear (National Diagnostics, HS-202) twice, 100% ethanol, 95% ethanol, and 70% ethanol, and gradually changed to dH2O. Antigen retrieval was performed by immersing the slides in Tris-EDTA buffer and incubating them in a high-pressure cooker for 7 min, followed by 20-min incubation at room temperature and pressure. After washing in water and PBS, the slides were blocked for 1 h in Protein Block (Dako, X0909) and incubated in primary antibody mix overnight at 4°C. If using mouse primary antibodies, anti-mouse Fab fragments were added in the primary antibody mix at 1:10 (Jackson ImmunoResearch, 715-007-003). The slides were then washed in PBS and PBST and incubated in secondary antibody mix at room temperature for 1 h 30 min. Slides were washed in PBS and PBST and mounted with Mowiol 4–88 (Sigma-Aldrich 9002-89-5) and left to cure overnight. Primary antibodies used were as follows: chicken anti-KRT5 (1:500, Biolegend #905901), rabbit anti-KRT10 (1:100, Abcam, ab76318), guinea pig anti-KRT10 (1:100, Progen, GP-K10), mouse anti-Involucrin (1:100, Invitrogen, MA5-11803), rabbit anti-pAKT (1:100, Cell Signaling Technology, #4060), rat anti-Ki67 (1:200, eBioscience #14-5698-82), rat anti-pHH3 (1:200, Biolegend, #641002), rabbit anti-pHH3 (1:200, Millipore, #06-570), rabbit anti-gamma tubulin (1:100, Abcam, #ab11317). Secondary antibodies used were as follows: goat anti-chicken Dylight 405 (Rockland, #603-146-002), donkey anti-chicken CF633 (Sigma, #SAB4600127), goat anti-rabbit CF488 (Sigma, #SAB4600234), donkey anti-rabbit AF568 (Sigma, #SAB4600076), donkey anti-guinea pig CF405 (Sigma, #SAB4600467), goat anti-guinea pig AF647 (Molecular Probes, #A21450), goat anti-mouse AF488 (Invitrogen, #A11001), goat anti-rat AF488 (Cell Signaling Technology, #4416) and goat anti-rat AF555 (Cell Signaling Technology, #4417). All secondary antibodies were used at 1:500. TUNEL staining was done using the in situ Cell Death Detection Kit, TMR Red (Roche, #12156792910), following manufacturer’s protocol.

### Microscopy and image analysis

Sections stained with H&E were scanned on an Aperio XT Slide Scanner (Leica). Immunofluorescence images were acquired using either a Zeiss LSM710 or LSM800 confocal laser scanning microscope, equipped with a 20x/NA = 0.80 dry plan apochromat objective, from the McGill University Advanced BioImaging Facility (ABIF, RRID:SCR_017697). Image analyses were performed with ImageJ v1.53. Spindle angles were traced through both spindle poles and the basal layer was used as a reference. Rare events (TUNEL+ cells and Krt5- Ki67+ cells) were quantified by assessing the number of cells per μm of basement membrane to capture as many events as possible.

### Live-imaging

This protocol was adapted from live-imaging protocols for embryonic skin explants in Cetera *et al.* (2018) and Lough *et al.* (2019).

For all live-imaging experiments, we used Krt5^CreERT2^; R26^tdTomato^; H2B-GFP; LGN+/+ or LGN–/– mice aged between 9- and 24-wk-old. At least 24 h before the time of imaging, the mouse was injected intraperitoneally with a solution of 1 mg of tamoxifen dissolved in corn oil.

On the day of imaging, a solution of 3:1 DMEM:F12 and 1% agarose was heated to a boil and cooled to 50°C, before mixing in 1:10 FBS, 1:100 L-glutamine, 1:100 sodium bicarbonate, 1:100 sodium pyruvate, and 1:100 penicillin/streptomycin. The solution was poured in a 50-mm dish and left to cool and gel at room temperature.

Immediately after sacrifice, depilatory cream was applied for 3 min to remove the hairs on the lips and mystacial pads. After rinsing the cream with water, the lips and mystacial pads were dissected. The mystacial pads and the inner part of the lip were removed. Outer lip samples measuring about 0.8 mm × 0.4 mm were placed dermal side onto the gelled medium disk, and epidermal side in a 35-mm lumox dish (Sarstedt, 94.6077.331), taking care not to compress the tissue on the lumox membrane to prevent z-drift during imaging. Explants were cultured at 37°C in 5% CO_2_ for 30 min to 1 h before imaging, and throughout imaging. Imaging was performed with a Quorum WaveFX-X1 spinning disk confocal system, on a Leica DMI6000B inverted microscope, equipped with a 20x/NA = 0.7 objective. Images were acquired every 5 min for 13 to 15 h with a Z-series of 1.5–2 μm for a total range of 20–30 μm. Imaging was done away from the tissue edges.

### Statistical analysis

Statistics were performed using Prism 9.0 software (Graphpad). All spindle angle distributions were tested using the Kolmogorov-Smirnov test. For the rest of the data, normality was tested with the Shapiro-Wilk normality test. For normal data, statistical comparisons of two conditions were done using unpaired two-tailed Student’s *t* test, and statistical comparisons of three or more conditions were done using one-way ANOVA. For non-normal data, statistical comparisons of two conditions were done using two-tailed Mann-Whitney test. The randomization scores were computed using Python. To establish the randomization scores, we compared each experimental distribution to 100,000 random distributions of the same size and performed a Kolmogorov-Smirnov test to compare the distributions. We compiled a randomization score by taking the percentage of *p* values higher than α (α = 0.05, statistical significance, or α = 0.01, high statistical significance). The randomization score represents the likelihood that the experimental distribution is statistically equivalent to a random distribution. The outlier test (Figure 4G) was performed by generating a Gaussian distribution using the 14d PTEN LGN^+/+^ mean and SD. Six values were then randomly generated from that distribution 10,000 times. The *p* value was the likelihood that one of those values would be above 0.0007. The Python scripts are available upon request.

## Supplementary Material





## Abbreviations used:

DMEM: Dulbecco’s Modified Eagle Medium
EDTA: ethylenediaminetetraacetic acid
FACS: fluorescence-activated cell sorting
FBS: fetal bovine serum
H&E: hematoxylin and eosin
PBS: phosphate buffered saline
PBST: phosphate buffered saline-Tween 20
PCR: polymerase chain reaction
PFA: paraformaldehyde
rcf: relative centrifugal force
TUNEL: terminal deoxynucleotidyl transferase dUTP nick end labeling
