# Supplementary material for: LGN loss randomizes spindle orientation and accelerates tumorigenesis in PTEN-deficient epidermis
Source: Mol Biol Cell. 2024 Jan 12;35(2):br5. doi: 10.1091/mbc.E23-03-0111 (PMC10881154; doi:10.1091/mbc.E23-03-0111)
Supplement: Supplementary file 1 [file mbc-35-br5-s001.pdf]

Supplementary Materials

Molecular Biology of the Cell

Viala *et al*.

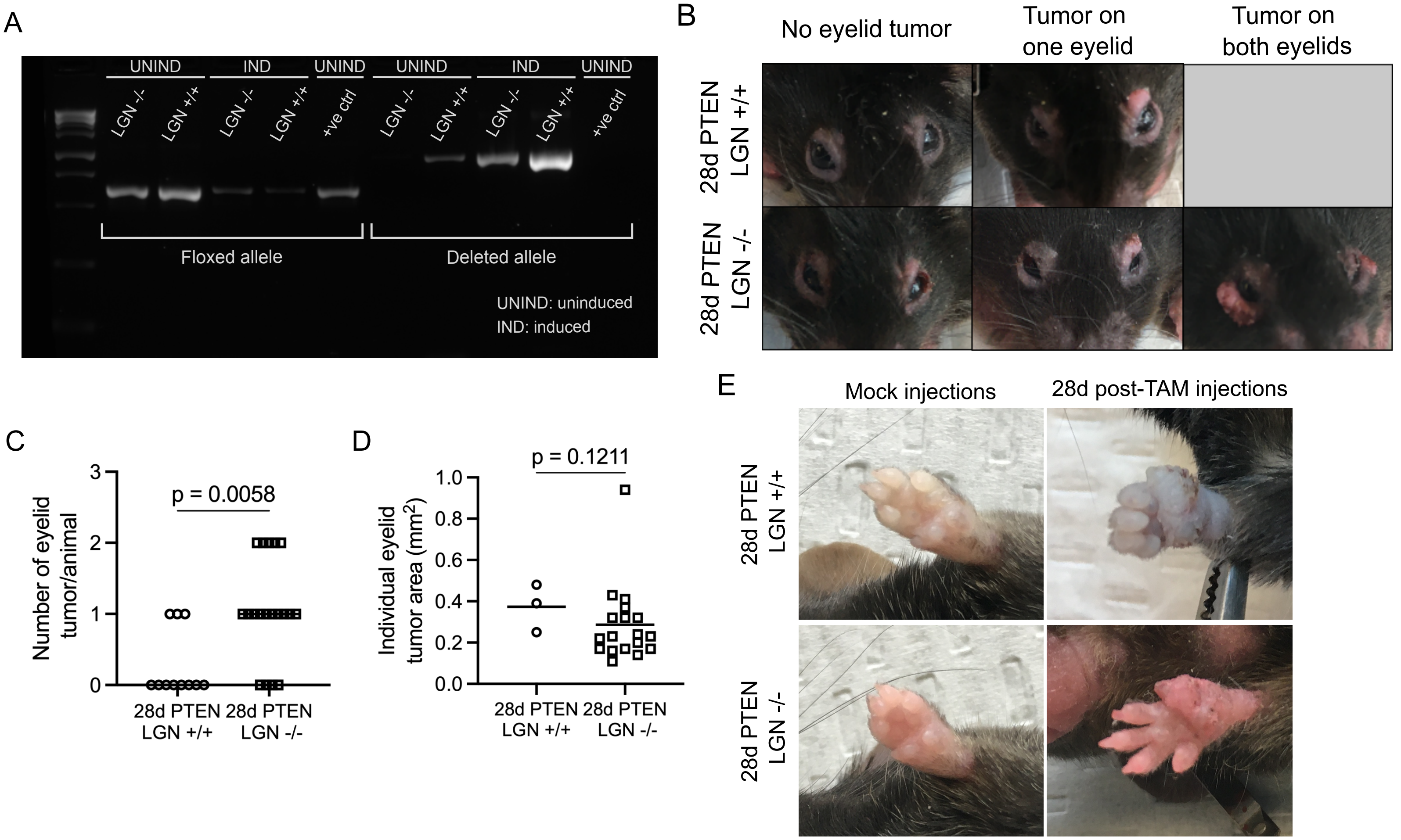

Figure S1. (A) PCR genotyping for the floxed and deleted PTEN alleles. PTEN LGN+/+ (LGN +/+) and PTEN LGN-/- (LGN -/-) mice were injected with tamoxifen (induced) or corn oil (uninduced) and after 21 days, basal cells from the footpad epidermis were isolated and genotyped. The positive control (+ve ctrl) is pooled from uninduced LGN+/+ and LGN-/- mice. N= 3 mice for all conditions except induced PTEN LGN+/+, which is n=1. (B) Representative images of the face, showing the eyelids. (C) Quantification of eyelid tumor distribution. N = 11 mice for 28d PTEN LGN +/+ and n = 19 mice for 28d PTEN LGN -/- tested with two-tailed Mann-Whitney test. (D) Quantification of individual eyelid tumor area. N = 3 tumors from 3 mice for K5 PTEN LGN +/+ and n = 18 tumors from 12 mice for K5 PTEN LGN -/-, tested with two-tailed Mann-Whitney test. (E) Representative images of a paw of PTEN LGN +/+ and PTEN LGN -/- mice, 28 days after mock injections or tamoxifen injections.

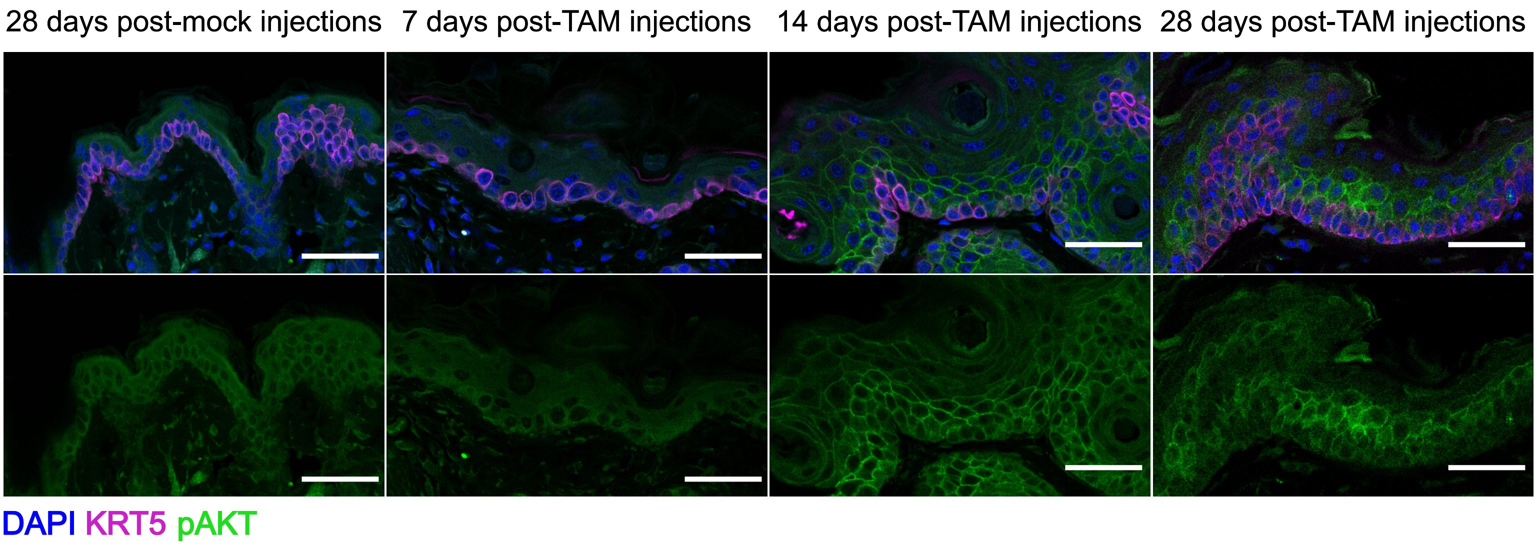

Figure S2. Immunostaining of K5 PTEN LGN +/+ snout epithelium 28 days after mock injections, 7 days post-tamoxifen (TAM) injections, 14 days post-TAM injections and 28 days post-TAM injections, marked with DAPI, KRT5 and pAKT. Scale bar = 50 μm.

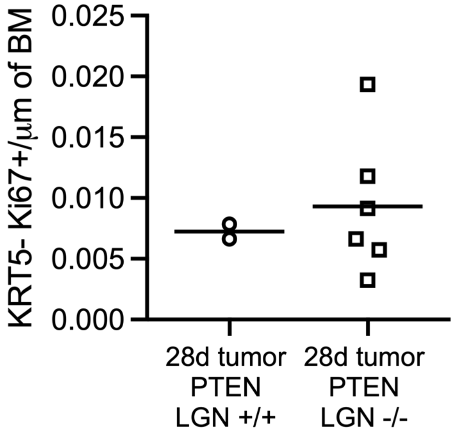

Figure S3. Frequency of proliferative spinous cells in 28d PTEN LGN +/+ and LGN -/- snout tumors. N= 2 mice for 28d PTEN LGN +/+ and n = 6 mice for 28d PTEN LGN -/-, >2400 μm/animal.
