# Supplementary material for: LGN loss randomizes spindle orientation and accelerates tumorigenesis in PTEN-deficient epidermis
Source: Mol Biol Cell. 2024 Jan 12;35(2):br5. doi: 10.1091/mbc.E23-03-0111 (PMC10881154; doi:10.1091/mbc.E23-03-0111)
Supplement: Supplementary file 2 [file mbc-35-br5-s002.pdf]

LGN loss randomizes spindle orientation and accelerates tumorigenesis in PTEN-deficient epidermis

Sophie Viala, Charlotte Hadjadj, Vandana Nathan, Marie-Christine Guiot, Luke McCaffrey, Katie Cockburn, and Maxime Bouchard

Corresponding author(s): Sophie Viala, McGill University

Review Timeline:

Submission Date:	2023-03-30
Editorial Decision:	2023-04-24
Revision Received:	2023-10-01
Editorial Decision:	2023-10-23
Revision Received:	2023-11-03
Accepted:	2023-11-07

Editor-in-Chief: Matthew Welch

Transaction Report:

(Note: With the exception of the correction of typographical or spelling errors that could be a source of ambiguity, letters and reports are not edited. The original formatting of letters and referee reports may not be reflected in this compilation.)

RE: Manuscript #E23-03-0111
TITLE: Randomized spindle orientation accelerates tumorigenesis in PTEN-deficient epidermis

Dear Dr. Viala:

Two reviewers have provided comments on your manuscript. While they demonstrated interest in the phenotypes observed, they have significant concerns with both the analysis and presentation of data, and have raised some questions about the strength of conclusions and the advancement to the field. That said, it is likely that additional experiments, as well as changes to the figure layout and text will be required for acceptance.
Should you decide to try to address these concerns, please also ensure that you adhere to the Author Submission checklist and that all required information is contained in the revised manuscript.

Sincerely,

Terry Lechler
Monitoring Editor
Molecular Biology of the Cell

Dear Dr. Viala,

The review of your manuscript, referenced above, is now complete. The Monitoring Editor has decided that your manuscript is not acceptable for publication at this time, but may be deemed acceptable after specific revisions are made, as described in the Monitoring Editor's decision letter above and the reviewer comments below.

A reminder: Please do not contact the Monitoring Editor directly regarding your manuscript. If you have any questions regarding the review process or the decision, please contact the MBoC Editorial Office (mboc@ascb.org).

When submitting your revision include a rebuttal letter that details, point-by-point, how the Monitoring Editor's and reviewers' comments have been addressed. (The file type for this letter must be "rebuttal letter"; do not include your response to the Monitoring Editor and reviewers in a "cover letter.") Please bear in mind that your rebuttal letter will be published with your paper if it is accepted, unless you have opted out of publishing the review history.

Authors are allowed 90 days to submit a revision. If this time period is inadequate, please contact us at mboc@ascb.org.

Revised manuscripts are assigned to the original Monitoring Editor whenever possible. However, special circumstances may preclude this. Also, revised manuscripts are often sent out for re-review, usually to the original reviewers when possible. The Monitoring Editor may solicit additional reviews if it is deemed necessary to render a completely informed decision.

In preparing your revised manuscript, please follow the instruction in the Information for Authors (www.molbiolcell.org/info-for-authors). In particular, to prepare for the possible acceptance of your revised manuscript, submit final, publication-quality figures with your revision as described.

To submit the rebuttal letter, revised manuscript, and figures, use this link: Link Not Available

MBoC offers the option to publish your work with immediate open access. Open access can increase the discoverability and usability of your research. If you would like to publish your paper with immediate open access but did not select this option during initial submission, please contact the editorial office at mbc@ascb.org.

Thank you for submitting your manuscript to MBoC. We look forward to receiving your revised paper.

Sincerely,

Eric Baker
Journal Production Manager
MBoC Editorial Office
mbc@ascb.org

Reviewer #1 (Remarks to the Author):

This manuscript addresses whether spindle orientation promotes skin tumorigenesis. The authors combine a skin cancer-promoting PTEN genetic model with an LGN deletion previously established to dysregulate spindle orientation. A similar question was addressed by Morrow et al. eLife 2019; Viala et al. utilizes a distinct cancer and spindle misorientation mouse model combination to support the claims of the published work. This unfortunately compromises the novelty of the current study. Furthermore, the mechanism of how an LGN spindle orientation mutant promotes tumorigenesis in the PTEN mutant background is not thoroughly flushed out in this manuscript. The authors performed very thorough quantifications but reveal no clear impact of LGN-driven spindle misorientation on epidermal cell behaviors or tissue architecture in the PTEN mutant background; no significant aberrances in differentiation, apoptosis, or proliferation were noted that could account for the accelerated tumorigenesis observed. This manuscript would benefit from better explanations/interpretation of the findings. It is ultimately unclear how this work moves the field forward or provides additional insights into how spindle orientation drives tumorigenesis.

Morrow et al. 2019 eLife used a NuMA mutation that caused spindle misorientation by perturbing NuMA-microtubule interactions, not a "loss of NuMA" as stated in the manuscript.

The claim that "LGN loss accelerates epidermal tumor growth after PTEN deletion" suggests that the PTEN mutant mice would have an equal number of tumors and tumor burden compared with the double mutant if the timeline were to be extended...is this true, or is the combination of PTEN and LGN mutations more tumorigenic?

The term "several epithelia" in the last sentence before the section break on page 5 is misleading - "distinct anatomical skin regions" would be more accurate.

The authors do not explain why LGN loss alters spindle orientation without impacting architecture. Is telophase rescue occurring?

During which mitotic stage(s) were spindle angles scored? It appears in Figure 2E that spindles were scored during metaphase, although anaphase would be a more appropriate stage since this is when spindle orientation is likely to be locked in. A channel showing DNA in the spindles would be helpful and using more distinct colors than red/pink for the y-tubulin and KRT5 channels would make these proteins more easily distinguishable in the images.

Where is LGN localized in adult snout epidermis? At the basolateral junctions? Why do the authors believe LGN is required for parallel basal spindle orientations in adult skin vs its clear apical localization and facilitation of perpendicular divisions in embryonic skin? How do the authors propose that LGN is driving parallel divisions in their model?

Wouldn't the authors expect basal layer density to be significantly altered by the increased perpendicular divisions at the expense of parallel divisions in LGN mutant epidermis? How do the authors interpret LGN deletion to have negligible impact on tissue architecture and differentiation? The authors need to spell out for the reader how they believe the observed spindle orientation defects are causing enhanced tumorigenesis in their PTEN/LGN combination model. This explanation must then be substantiated by further experimentation and analyses to support their rationale.

Reviewer #2 (Remarks to the Author):

In this manuscript the authors investigated the effect of spindle orientation on mouse skin tumorigenesis by studying basal epithelial-specific PTEN deficient mouse model in WT or LGN knockout background. They found that LGN loss worsens the development of papillomas on mouse snout and eyelid. They further showed that LGN loss mainly impacts spindle orientations by shifting from planar to perpendicular divisions in both WT or PTEN deficient background. The manuscript is well-written and the experiments are clearly laid out. However, the conclusions could be strengthened through changes in data analyses/presentations as laid out below.

Major comments:

1) Figure 1H, it would be better if the authors have quantifications/statistical comparisons (a graph of percentage of lower and higher grade/mixed tissue architecture type of papillomas in LGN knockout vs. WT mice) in addition to the representative picture.

2) It is unclear how efficient PTEN knockout/Cre recombination is. I assume it is probably close to 100%, but it would be nice if the authors could validate it, for example though genotyping for recombined vs. WT alleles. Related, In Figure S3, pAKT is higher in the intermediate suprabasal vs. basal cells; could it be that PTEN knockout cells have differentiated/delaminated and the pAKT negative WT cells have compensated for the loss by replacing the PTEN mutant cell areas in the basal compartment? A heterogeneously activated tissue would influence the quantifications for supposedly PTEN mutant tissues.

3) For comparison of PTEN WT and deficient conditions, the authors referenced panels in figure 2 and figure 3, e.g. 2B and 3B, 2C and 3C for cell density measurement, 2F and 3E, 2G and 3F for spindle orientation, 2H and 3G for K10+ cells in basal layer, 2K and 3J for proliferation. It would be better to plot them side by side so statistical analyses can be done and readers could see the difference in the same graph, instead of simply saying, for example, by looking at figures 2B and 3B, cell density in LGN+/+ PTEN-/- vs PTEN +/+ increased by 31%.

4) Continuing with the previous point, if the authors lay 2F and 3E or 2G and 3F side by side, it might become apparent that loss of PTEN is significantly shifting the spindle orientation from planar to perpendicular in LGN WT condition, albeit not to the same degree as that caused by LGN loss. In this case the statement "PTEN loss is not sufficient to disrupt spindle orientation" and in the discussion "in the snout, spindle orientation is not significantly impacted by the loss of PTEN" would need to be revised.

5) Fig 2K and 3J, in the LGN-/- condition PTEN-/- mice have significantly lower percentage of pHH3+ basal cells compared to PTEN+/+ mice, does this mean the increased proliferation caused by LGN loss alone is negated by PTEN loss? It is possible the proliferation rate at Day 14 post tam treatment does not reflect increased proliferation in earlier phase. Could the authors comment on this?

6) The conclusion: "neither proliferation nor differentiation are affected by the combined loss of LGN and PTEN" cannot be made with the current layout of figure 2 and 3, in which WT condition is not directly compared to PTEN-/- LGN-/- condition.

7) The conclusion that LGN loss does not influence cell density/tissue folding is not fully convincing. In panel 2B, C, D and 3B, C, D, the average for LGN-/- condition is always higher than LGN+/+ condition, and I suspect the p value might approach being statistically significant if more mice are included, suggesting LGN could promote expansion of both basal and suprabasal compartments in both WT and PTEN deficient conditions.

8) The conclusion that "spindle defects are sufficient to accelerate the onset of tumorigenesis" is too strong without completely ruling out the effect of LGN on proliferation (the point above).

9) The statement in the title "Randomized spindle orientation accelerates tumorigenesis in PTEN deficient epidermis" is too strong. The relationship between spindle orientation and tumorigenesis depicted in the manuscript is correlative, not causative.

Minor points:

1) Figure 1E, according to the legend, is documenting "total tumor volume", however in the text the authors are referring to the "largest tumor of each genotype, with a 110-fold difference in size...", I realize the authors are referring to the highest dot in 1E, which is probably from a mouse with a single tumor so "total tumor volume" happens to be the volume for the single biggest tumor. To make the statement in the text an extra graph showing sizes of individual tumors would be helpful.

2) An additional graph for tumor burden of eyelid tumors like that for snout tumors would be informative.

3) Figure S2 only has representative images and no quantifications/statistical analysis.

4) Some images are of poor quality. Specifically, in figure 1F the bottom right picture of eyelid tumors on a mouse appears to be out of focus.

5) I'm curious as to why development of tumor on paws is independent of LGN status. Does it have anything to do with the paws being more stratified and has a higher percentage of perpendicular division at baseline?

6) Please expand on how LGN promotes planar division mechanistically in the discussion.

7) In the "Morrow et al., 2019" paper cited in the manuscript, loss of NuMA leads to more planar divisions that promote tumorigenesis by Kras mutant in skin, which is opposite to what the authors observed: more perpendicular, not planar divisions, promote tumorigenesis. The authors said in the discussion that this happens through "local accumulation of suprabasal cells". It is unclear how accumulation of differentiated cells would accelerate tumor development in the sense of pushing transitioning of papilloma to carcinoma. Please expand on this in the discussion.

We thank the reviewers for their attentive reading of this manuscript and for their thoughtful comments and questions, which have significantly improved this work. A list of changes is included on the first page of the manuscript for the reviewers' convenience.

Reviewer #1 (Remarks to the Author):

This manuscript addresses whether spindle orientation promotes skin tumorigenesis. The authors combine a skin cancer-promoting PTEN genetic model with an LGN deletion previously established to dysregulate spindle orientation. A similar question was addressed by Morrow et al. eLife 2019; Viala et al. utilizes a distinct cancer and spindle misorientation mouse model combination to support the claims of the published work. This unfortunately compromises the novelty of the current study.

We agree that this study builds on the understanding established by Morrow et al, however we believe that our data reveals additional novel insights into the relationship between spindle orientation and tumor formation.

Morrow et al (2019) show an increase in perpendicular divisions in basal cells of the footpad epidermis upon KRAS activation. When it is combined with disruption of NuMA function however, the increase in perpendicular divisions disappears, leading to massive tissue overgrowth. In contrast to these findings, we have discovered that PTEN deletion in snout epidermis does not markedly increase perpendicular divisions. Moreover, the combination of LGN and PTEN deficiency increases the proportion of oblique and perpendicular divisions, contrary to the phenotype observed with disruption of NuMA. Thus, unlike the data shown in Morrow et al, expansion of the basal stem cell pool is not what drives tumorigenesis LGN; PTEN double mutant tissue. In the revised version of this manuscript, we have more closely examined the hyperplastic PTEN mutant epithelium at 14d post-tamoxifen injection and have observed rare proliferative spinous cells in the suprabasal layers (Revised Figure 4 F&G). These cells appear more frequently in a subset of PTEN; LGN-/- animals and are common in 28d tumor tissue. We propose that the higher number of misoriented oblique/perpendicular divisions in PTEN; LGN-/- epidermis may contribute to tumor initiation at least in part by giving rise to spinous cells which retain the ability to proliferate.

Our work therefore reveals that the involvement of different epidermal regions (footpad vs snout), spindle regulators (NuMA vs LGN), and oncogenic models (KRAS activation vs PTEN deletion) can synergize in unique ways. This study emphasizes the need for context-specific understanding of mechanisms of tumorigenesis.

Furthermore, the mechanism of how an LGN spindle orientation mutant promotes tumorigenesis in the PTEN mutant background is not thoroughly flushed out in this manuscript. The authors performed very thorough quantifications but reveal no clear impact of LGN-driven spindle misorientation on epidermal cell behaviors or tissue architecture in the PTEN mutant background; no significant aberrances in differentiation, apoptosis, or proliferation were noted that could account for the accelerated tumorigenesis observed. This manuscript would benefit from better explanations/interpretation of the findings. It is ultimately unclear how this work moves the field forward or provides additional insights into how spindle orientation drives tumorigenesis.

We agree that gaining a better understanding of the cellular mechanisms underlying accelerated tumorigenesis in LGN mutant tissue is important. In our revised manuscript, we have examined proliferative behaviours more closely in the PTEN LGN mutant tissue and discovered the existence of rare spinous (Krt5-) proliferative (Ki67+) cells. These cells were more frequent in a subset of PTEN LGN mutant animals, and commonly occurring in tumor tissue (revised Figure 4, F and G). As discussed above, we propose that misoriented divisions increase the likelihood of generating these proliferative spinous cells, which in turn may contribute to tumor initiation. We therefore believe that

our initial novel observation that increased oblique/perpendicular divisions and contribution to tumorigenesis, together with this newly added observation of suprabasal proliferative cells as a potential mechanism, contributes new insight to the question of how spindle orientation impacts tumor formation.

Morrow et al. 2019 eLife used a NuMA mutation that caused spindle misorientation by perturbing NuMA-microtubule interactions, not a "loss of NuMA" as stated in the manuscript.

We thank the reviewer for noticing this, the text has been corrected (highlighted, pages 3 and 11).

The claim that "LGN loss accelerates epidermal tumor growth after PTEN deletion" suggests that the PTEN mutant mice would have an equal number of tumors and tumor burden compared with the double mutant if the timeline were to be extended...is this true, or is the combination of PTEN and LGN mutations more tumorigenic?

We thank the reviewer for this question. Our results show that loss of LGN accelerates tumor initiation, but we cannot draw conclusions on whether LGN also impacts the growth of tumors once they have formed. At 28d post-injection, 35% of PTEN; LGN -/- mice have tumors, compared to 12% in PTEN; LGN +/+, indicating that tumors arise more frequently in LGN -/- mice. While the LGN KO tumors are much larger than the LGN +/+ ones, we cannot say whether this is due to faster tumor growth or earlier tumor initiation. PTEN; LGN+/+ mutants do eventually develop tumors at later time-points. We kept a group of 5 K5CrERT2; PTENflox/flox; LGN +/+ mice for 56 days post-injection instead of 28 days post-injection. One mouse developed a tumor similar to the one observed in the LGN-/- group (see picture below), suggesting that LGN +/+ tumors can grow to resemble LGN -/- ones. This observation is in line with a previous study which found that all Krt5Cre; Ptenflox/flox mice eventually develop epidermal tumors (mostly squamous papillomas) between 3.5 and 8.5 months of age (Suzuki et al., 2003).

Together, this indicates that PTEN loss alone eventually leads to tumor formation and that, in this context, loss of LGN impacts the kinetics of tumor initiation. While there is some evidence that LGN +/+ can reach similar sizes to LGN -/- tumors, we cannot make conclusions on the role of LGN in tumor growth. We have corrected the manuscript to make this distinction more clear (highlighted, pp.4, 5, 6).

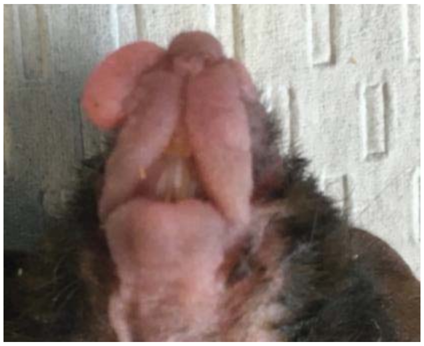

The term "several epithelia" in the last sentence before the section break on page 5 is misleading - "distinct anatomical skin regions" would be more accurate.

We thank the reviewer for this suggestion. It has been changed in the text (highlighted, page 5).

The authors do not explain why LGN loss alters spindle orientation without impacting architecture. Is telophase rescue occurring?

We thank the reviewer for this question. Several processes could explain the normal tissue architecture in the LGN mutant animals. The spindle orientation defects could be resolved by apoptosis of misplaced cells, telophase rescue/correction, daughter cells later reintegrating the basal layer, or a decrease in the rate of differentiation. We checked for apoptosis using a TUNEL assay and in response to a request from Reviewer 2, we have now quantified cell death (see below and revised Figure 4, H and I). We did not observe significant differences between the LGN wildtype and the LGN mutant. We also did not find differences in differentiation rates (revised Figure 4B).

To assess whether correction during telophase might explain the normal architecture of the LGN mutant tissue, we optimized and performed live-imaging experiments of adult skin explants, by adapting the protocols developed in Cetera et al. (2018) and Lough et al. (2019). To visualize basal divisions in the epidermis and track daughter cells, we crossed Krt5CreERT2; LGN +/+ or LGN-/- mice with a R26tdTomato; H2B-GFP mouse line. In the resulting mice, chromosomes are labelled with GFP and tdTomato expression can be used to mark the epidermis and identify cells in the basal layer (see revised Figure 3A). We first measured the angle at onset of anaphase (first frame of anaphase) to assess whether it recapitulates our result in tissue sections at metaphase. We found a similar division angle distribution in this assay; the majority of LGN +/+ cells start anaphase in a planar orientation (95% of divisions under 20°) while LGN -/- dividing cells have a much wider distribution of angles (66% of divisions under 20°) (see revised Figure 3, B and C).

In embryonic epidermis, oblique divisions can occur at metaphase and anaphase but reorient to either parallel or perpendicular divisions during telophase (Lough et al., 2019). To assess whether telophase correction is happening in our system, we measured the angle of division 1 hour after the onset of anaphase in the same population of cells. We found that most oblique divisions do not resolve within 1 hour of anaphase, which indicates that telophase correction is not the primary mechanism that rectifies aberrant divisions (revised Figure 3, D and E). However, we were able to track a subset of daughter cells from these oblique divisions over longer periods of time, and we found that some of these suprabasal cells can reintegrate into the basal layer (revised Figure 3F). It took up to 6 hours after onset of anaphase for the suprabasally-positioned daughter cell to move back into the basal layer. This phenomenon was not observed for all cells, whether because we did not image for long enough, or because it does not happen in every situation. We hypothesize that this ability of daughter cells to reintegrate in the basal layer, even hours after the end of the division, may at least in part account for the lack of defects in tissue architecture in LGN mutants. Altogether, our newly added explant imaging data showcases the resilience of the tissue in the context of spindle orientation errors.

Performing the same imaging experiments on K5CreERT2; PTENflox/flox; LGN+/+ or LGN-/-; R26tdTomato; H2BGFP mice was unfortunately not possible in the time provided for revisions due to the additional generations of breeding required, but we nonetheless believe that our newly added time-lapse imaging provides important new insights into the relationship between spindle orientation and epidermal homeostasis.

During which mitotic stage(s) were spindle angles scored? It appears in Figure 2E that spindles were scored during metaphase, although anaphase would be a more appropriate stage since this is when spindle orientation is likely to be locked in. A channel showing DNA in the spindles would be helpful and using more distinct colors than red/pink for the y-tubulin and KRT5 channels would make these proteins more easily distinguishable in the images.

We thank the reviewer for this question. The majority of spindle angles were indeed scored at metaphase, as we could find very few cells in anaphase or telophase. To get a more accurate assessment of spindle orientation, we performed live-imaging experiments (see comment above). We measured the angle of divisions at onset of anaphase and found similar patterns to the ones we had obtained when measuring angles on sections; the majority of LGN+/+ cells start anaphase in a

planar orientation (mean 6.2°; 95% of divisions under 20°) while LGN-/- dividing cells have a much wider distribution of angles (mean 17°; 66% of divisions under 20°). Interestingly, no division angles above 50° were observed in our live imaging data, in contrast to our observations from fixed tissue. This could be a reflection of changes in spindle orientation between metaphase and anaphase, as the spindle gets firmly anchored, as suggested. Improved accuracy of angle measurement with live-imaging could also contribute to this difference. Regardless, our newly added live imaging data supports our initial conclusion that LGN-/- cells are more likely to divide in an oblique fashion.

Figure 2 has also been modified to improve visualization of the DNA and the spindle poles by changing the colors and adding a separate image to show DNA and spindle poles alone (revised Figure 2B).

Where is LGN localized in adult snout epidermis? At the basolateral junctions? Why do the authors believe LGN is required for parallel basal spindle orientations in adult skin vs its clear apical localization and facilitation of perpendicular divisions in embryonic skin? How do the authors propose that LGN is driving parallel divisions in their model?

We thank the reviewer for these questions. We did attempt to visualize LGN localization using immunofluorescence. Staining for LGN, however, is notoriously difficult, and we were not able to detect LGN in sections of this tissue. Our interpretation of the localization and role of LGN here is therefore based on the changes observed when it is lost. During embryonic development, basal cells need to undergo perpendicular divisions for proper stratification of the skin. The core spindle orientation machinery is responsible for regulating the perpendicular orientation of dividing basal cells (Williams et al., 2011; Williams et al., 2014). This paradigm changes in adulthood however, when the majority of basal cells need to maintain a planar orientation during mitosis (Ipponjima et al., 2016; Smart, 1970). It is likely that the core machinery responsible for the regulation of spindle orientation in the embryo is also involved in the maintenance of these planar divisions in adulthood.

We propose that LGN localizes to the basolateral cortex, and through its canonical interactions with NuMA and Gai, participates in the anchoring of the spindle poles. While we do not think that LGN is required for planar divisions (we find that more than half of divisions in LGN -/- are planar in both stained sections and live-imaging), we think that LGN participates in the maintenance of planar divisions.

There are a couple of ways that a change in LGN localization from apical to basolateral could be achieved. In embryonic skin, Insc has been shown to recruit LGN apically to promote perpendicular divisions (Williams et al, 2014). In adult tissue however, single-cell RNA-sequencing evidence suggests that, unlike LGN, Insc is not expressed in proliferating basal cells (Joost et al., 2020) (see Reviewer Figure 1). In the absence of Insc, there may not be a way for LGN to be recruited to the apical domain. Conversely, SAPCD2, which has been shown to exclude LGN from the apical domain in mouse retinal progenitor cells (Chiu et al., 2016), is expressed in dividing basal cells of the adult epidermis (Joost et al., 2020). If SAPCD2 carries out a similar function in epidermal cells, it provides a mechanism for ensuring LGN localization at the basolateral cortex.

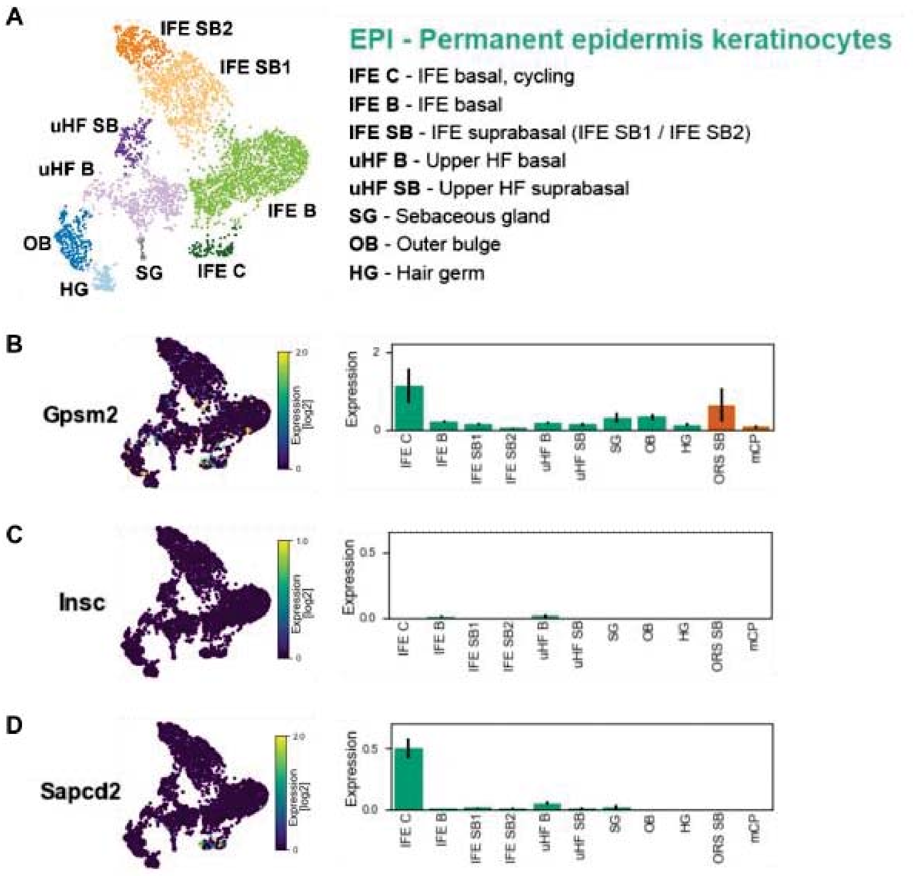

Reviewer Figure 1. Expression of spindle orientation regulators in adult epidermis. Data generated from Kasper lab's single-cell RNA sequencing of adult epidermis dataset using the online tool (http://kasperlab.org/tools, Joost et al. (2020)). (A) UMAP of the keratinocytes of the interfollicular epidermis, colored by cluster, including a cluster of proliferative basal cells, IFE C. (B-D) Expression of Gpsm2 (LGN) (B), Insc (C) and Sapcd2 (D), overlayed on the UMAP (left) and displayed on a bar graph (right).

Wouldn't the authors expect basal layer density to be significantly altered by the increased perpendicular divisions at the expense of parallel divisions in LGN mutant epidermis? How do the authors interpret LGN deletion to have negligible impact on tissue architecture and differentiation?

We agree that an increase in perpendicular divisions should result in altered tissue architecture. To understand why LGN mutant tissue appears normal, we optimized and performed live-imaging experiments which allows us to visualize dividing cells and track the outcomes of daughter cells. We found that suprabasally-positioned cells generated by oblique divisions can reintegrate the basal layers hours after the end of mitosis. This provides a mechanism for correcting aberrant positioning of daughter cells and maintaining a normal tissue architecture.

The authors need to spell out for the reader how they believe the observed spindle orientation defects are causing enhanced tumorigenesis in their PTEN/LGN combination model. This explanation must then be substantiated by further experimentation and analyses to support their rationale.

While deletion of PTEN in the basal layer leads to tumor growth in the snout epidermis, the delay between loss of PTEN and tumor initiation is highly variable. Suzuki et al (2003) report a 5-month range in timing of epidermal tumor initiation. Although we have not conducted long-term studies, we have observed a similar variability in the timing of tumor initiation, with some LGN-/- mice forming tumors as early as 15 days post-tamoxifen injections and others staying snout tumor-free for up to 2 months. This considerable temporal variability suggests that while the PTEN-deficient epidermis is primed for tumor initiation, additional, more rare (local) events or changes are likely needed to kick-start tumor initiation. We hypothesize that spindle orientation defects increase the probability of these events/changes occurring in the tissue, which then translates to an acceleration of tumor initiation. In addition, since about a third of PTEN LGN -/- mice develop snout tumors by 28d, we do not expect to see changes in all of these mice at 14d. Rather, we expect a subset of these mice to exhibit changes at 14d and go on to develop tumors by 28d.

To further address changes that could promote tumor initiation, we took a closer look at the 14d epidermis and found rare proliferative cells (Ki67+) that do not express Krt5 in the suprabasal layers of the epidermis. Furthermore, we observed a subset of mice in the PTEN LGN -/- group with an elevated proportion of these Krt5- Ki67+ cells. To check whether these aberrant cells are associated with tumors, we analyzed tissue from 28d tumors and found that these cells are more common in that tissue, confirming the association. We propose that misoriented divisions in PTEN LGN-/- epidermis give rise to spinous cells, which do not or cannot reintegrate the basal layer and retain the ability to proliferate, contributing to tumor initiation. The thickened basal layer in PTEN-deficient epidermis may prevent suprabasally-positioned cells from making contact with the basement membrane and reintegrating their layer. We were not able to test this hypothesis because of the significant additional time required to generate the mouse lines that would be needed for live imaging (Krt5^{CreERT2}; PTEN$^{fl/fl}$; LGN+/+ or LGN-/- combined with R26^{tdTomato}; H2B-GFP). However, the fact that these proliferative spinous cells appear more frequently in a third of PTEN LGN-/- animals at 14d is consistent with our data showing that one third of mice have accelerated tumor formation and supports our hypothesis that only a subset of animals undergo the changes associated to tumor initiation at that stage.

Reviewer #2 (Remarks to the Author):

In this manuscript the authors investigated the effect of spindle orientation on mouse skin tumorigenesis by studying basal epithelial-specific PTEN deficient mouse model in WT or LGN knockout background. They found that LGN loss worsens the development of papillomas on mouse snout and eyelid. They further showed that LGN loss mainly impacts spindle orientations by shifting from planar to perpendicular divisions in both WT or PTEN deficient background. The manuscript is well-written and the experiments are clearly laid out. However, the conclusions could be strengthened through changes in data analyses/presentations as laid out below.

Major comments:

1) Figure 1H, it would be better if the authors have quantifications/statistical comparisons (a graph of percentage of lower and higher grade/mixed tissue architecture type of papillomas in LGN knockout vs. WT mice) in addition to the representative picture.

Thank you for this comment. We have added the quantifications of tumor architecture (revised Figure 1I) and have found that in all tumors, at least half of the tissue presents some level of cellular disorganization. In LGN KO tumors, we consistently find that 30-40% of the tumor retains a layered architecture while the remaining 60-70% of the tissue displays perturbed cellular organization. Although the small number of tumors that formed in PTEN LGN+/+ limited our ability to perform statistical comparisons, visual inspection indicated that PTEN LGN-/- and PTEN LGN+/+ tumors had similar amounts of cellular disorganization.

2) It is unclear how efficient PTEN knockout/Cre recombination is. I assume it is probably close to 100%, but it would be nice if the authors could validate it, for example though genotyping for recombined vs. WT alleles. Related, In Figure S3, pAKT is higher in the intermediate suprabasal vs. basal cells; could it be that PTEN knockout cells have differentiated/delaminated and the pAKT negative WT cells have compensated for the loss by replacing the PTEN mutant cell areas in the basal compartment? A heterogeneously activated tissue would influence the quantifications for supposedly PTEN mutant tissues.

Thank you for this comment. We agree with the reviewer that the efficiency of recombination is likely high. We injected Krt5^{CreERT2}; PTEN$^{fl/fl}$ mice with 6mg of Tamoxifen, which in our hands is typically sufficient to cause recombination in all CreERT2-expressing cells. However, each floxed allele differs in its efficiency of recombination, and mosaicism of the Krt5^{CreERT2} expression could also potentially occur. We attempted to sort basal cells from snout epidermis using FACS and genotype these cells directly to address this question but were unable to establish an isolation protocol that was compatible with the snout tissue. However, we believe that incomplete recombination is unlikely to explain the phenotypic differences we see between genotypes because we would expect PTEN wildtype “escaper” cells to occur with similar frequency in LGN +/+ and LGN -/- tissue. This therefore seems insufficient to explain the significantly higher tumor incidence in the LGN-/- animals. Moreover, we see similar activation levels of pAKT in PTEN LGN +/+ and PTEN LGN -/- tissue, consistent with uniform PTEN loss in both genotypes (Reviewer Figure 2).

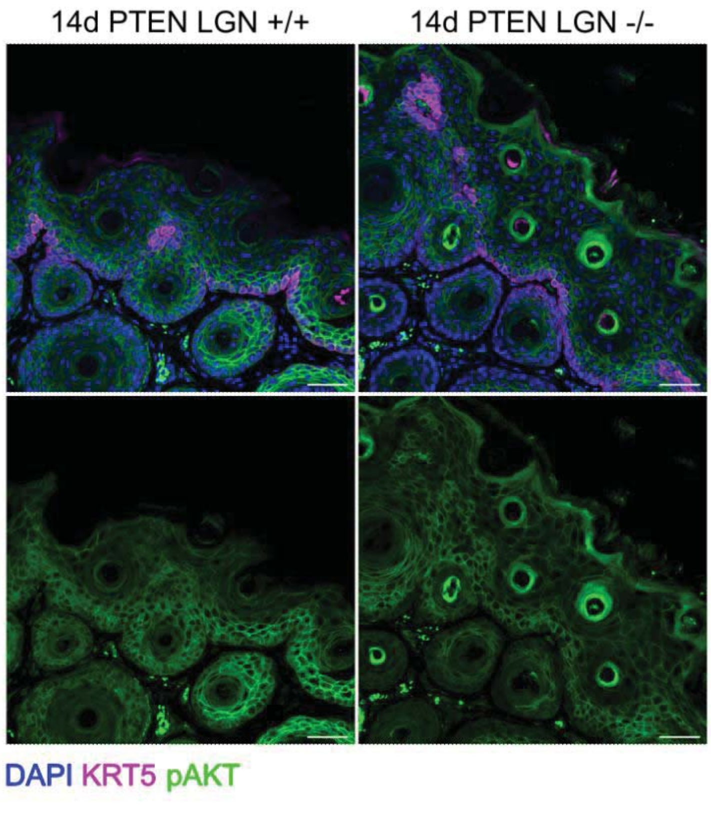

Reviewer Figure 2. Representative images of the snout epidermis at 14d in PTEN LGN +/+ and PTEN LGN -/- tissue, stained with DAPI, KRT5 and pAKT. Scale bar = 20 µm.

3) For comparison of PTEN WT and deficient conditions, the authors referenced panels in figure 2 and figure 3, e.g. 2B and 3B, 2C and 3C for cell density measurement, 2F and 3E, 2G and 3F for spindle orientation, 2H and 3G for K10+ cells in basal layer, 2K and 3J for proliferation. It would be

better to plot them side by side so statistical analyses can be done and readers could see the difference in the same graph, instead of simply saying, for example, by looking at figures 2B and 3B, cell density in LGN+/+ PTEN-/- vs PTEN +/+ increased by 31%.

We thank the reviewer for these recommendations to improve comparisons. We have updated figures as suggested (see revised Figures 2 and 4), to show the 4 conditions in the same graphs.

4) Continuing with the previous point, if the authors lay 2F and 3E or 2G and 3F side by side, it might become apparent that loss of PTEN is significantly shifting the spindle orientation from planar to perpendicular in LGN WT condition, albeit not to the same degree as that caused by LGN loss. In this case the statement "PTEN loss is not sufficient to disrupt spindle orientation" and in the discussion "in the snout, spindle orientation is not significantly impacted by the loss of PTEN" would need to be revised.

We have revised Figure 2 to combine the genotypes as suggested. When comparing spindle orientation in LGN+/+ vs PTEN LGN+/+ tissue, we observe that there is a trend towards increased perpendicular divisions, but this is not statistically significant (revised Figure 2C). We have change the text to soften the conclusion (highlighted, p.7).

5) Fig 2K and 3J, in the LGN-/- condition PTEN-/- mice have significantly lower percentage of pHH3+ basal cells compared to PTEN+/+ mice, does this mean the increased proliferation caused by LGN loss alone is negated by PTEN loss? It is possible the proliferation rate at Day 14 post tam treatment does not reflect increased proliferation in earlier phase. Could the authors comment on this?

We agree with the reviewer that the proliferation rate at 14d does not necessarily reflect changes in proliferation happening prior to that timepoint. Between day 7 and day 14 post-tamoxifen injections, the epithelium becomes hyperplastic, with marked increases in basal and spinous cell densities. This rapid expansion of both compartments must be supported by an increase in proliferation. The 14d stage may be the start of a temporary equilibrium following the rapid changes induced by loss of PTEN.

At 14d, the proliferation as measured by pHH3+ cells in PTEN mutants is not significantly different from the wildtype. It does trend down compared to the PTEN wildtype conditions. On the other hand, the number of Ki67+ cells trends upward. It could be that the increase in pHH3+ cells in LGN -/- tissue does reflect increased proliferation and that changes induced by PTEN loss override the effect of loss of LGN alone. However, we believe it is important to note that the proportion of pHH3+ cells could also be affected by the kinetics of mitosis itself. PTEN participates in the regulation of the mitosis and spindle assembly checkpoints and PTEN loss has been shown to accelerate mitosis in cultured cells in vitro (Choi et al., 2017; Liu et al., 2017). This downward trend in the proportion of pHH3+ cells in PTEN-deficient cells could reflect a faster cycling through mitosis.

6) The conclusion: "neither proliferation nor differentiation are affected by the combined loss of LGN and PTEN" cannot be made with the current layout of figure 2 and 3, in which WT condition is not directly compared to PTEN-/- LGN-/- condition.

We thank the reviewer for pointing this out. This statement was meant to express that at 14 days in PTEN-deficient conditions, loss of LGN does not impact proliferation or differentiation. We have changed the text to be more accurate (highlighted, p.11).

7) The conclusion that LGN loss does not influence cell density/tissue folding is not fully convincing. In panel 2B, C, D and 3B, C, D, the average for LGN-/- condition is always higher than LGN+/+ condition, and I suspect the p value might approach being statistically significant if more mice are

included, suggesting LGN could promote expansion of both basal and suprabasal compartments in both WT and PTEN deficient conditions.

We agree with the reviewer that there are trends in revised Figure 2 and 4 that point to LGN -/- tissue having higher cell density/folding. To assess whether there is a difference in absolute number of cells between the two groups, we combined the cell density and tissue folding measurements to determine the number of cells per length of the tissue (Reviewer Figure 3). Although the trends were maintained, there were no statistical differences between LGN+/+ and LGN-/- genotypes in either a wildtype or PTEN deficient background. Local changes may contribute to the variability in measurements. Despite the biological variability, the averages in cellular density of LGN+/+ and LGN-/- are very close to each other. For the basal layer, 16 cells/100µm in LGN +/+ vs 18 cells/100µm in LGN -/- and for the spinous layer, 19 cells/100µm for LGN +/+ and 20 cells/100µm for LGN -/- (revised Figure 2 F and G).

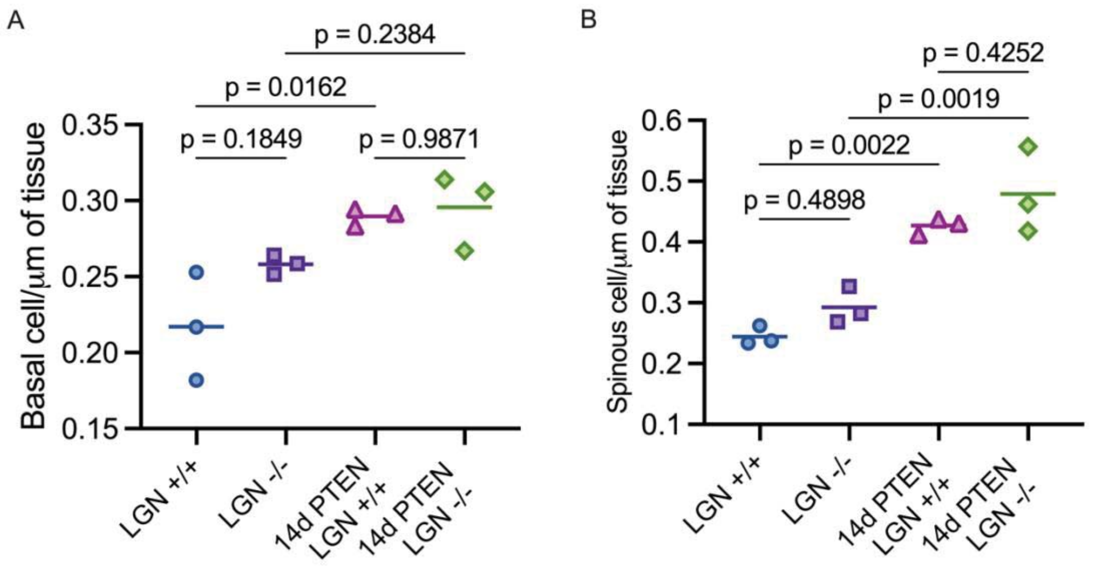

Reviewer Figure 3. Number of basal (A) or spinous (B) cells/length of tissue. These numbers were obtained by multiplying the cellular density by the folding measure to give a measure of the absolute number of cells per length of tissue.

8) The conclusion that "spindle defects are sufficient to accelerate the onset of tumorigenesis" is too strong without completely ruling out the effect of LGN on proliferation (the point above).

We thank the reviewer for this comment. We have changed the text to incorporate our new findings and provide a more nuanced statement (highlighted, p.12).

9) The statement in the title "Randomized spindle orientation accelerates tumorigenesis in PTEN deficient epidermis" is too strong. The relationship between spindle orientation and tumorigenesis depicted in the manuscript is correlative, not causative.

We have changed the title to “Loss of LGN randomizes spindle orientation and accelerates tumorigenesis in PTEN-deficient epidermis”.

Minor points:

1) Figure 1E, according to the legend, is documenting "total tumor volume", however in the text the

authors are referring to the "largest tumor of each genotype, with a 110-fold difference in size...", I realize the authors are referring to the highest dot in 1E, which is probably from a mouse with a single tumor so "total tumor volume" happens to be the volume for the single biggest tumor. To make the statement in the text an extra graph showing sizes of individual tumors would be helpful.

Thank you for pointing that out. We have now included a graph showing individual snout tumor size (newly added Figure 1G).

2) An additional graph for tumor burden of eyelid tumors like that for snout tumors would be informative.

We have now included a graph showing individual eyelid tumor area (newly added Figure S1C).

3) Figure S2 only has representative images and no quantifications/statistical analysis.

We have added quantifications in revised Figure 4I, which confirmed that cell death is rare in all conditions.

4) Some images are of poor quality. Specifically, in figure 1F the bottom right picture of eyelid tumors on a mouse appears to be out of focus.

We thank the reviewer for pointing this out and have provided a higher quality image in the revised manuscript. We have made sure to keep the quality of the images as we export the figures.

5) I'm curious as to why development of tumor on paws is independent of LGN status. Does it have anything to do with the paws being more stratified and has a higher percentage of perpendicular division at baseline?

We agree that footpad epidermis characteristics might explain why there are no difference between LGN wildtypes and LGN mutants in paw tumor formation at 28d. About a third of divisions in the adult footpad are non-planar during homeostasis (Ipponjima et al., 2016; Morrow et al., 2019). Disrupting spindle orientation in this tissue might therefore have a limited effect on tumor formation. In addition to this, tumors on the footpad developed earlier than tumors on the snout. By 14d post-injections, tumorigenesis had started on at least one paw in each animal. Because we used the 28d timepoint to count and measure tumors on the animals, we could be missing an effect of LGN at earlier time-points on paw tumor initiation specifically.

6) Please expand on how LGN promotes planar division mechanistically in the discussion.

We have updated the discussion to more clearly articulate mechanistically how LGN promotes planar divisions (highlighted, pp. 11-12). We propose that LGN localizes to the basolateral cortex, and through its canonical interactions with NuMA and Gai, participates in the anchoring of the spindle poles. While LGN has been shown to localize apically to enable perpendicular divisions in embryonic development (Williams et al., 2011; Williams et al., 2014), adult basal cells overwhelmingly divide to maintain planar divisions (Ipponjima et al., 2016; Smart, 1970).
As discussed above in our response to Reviewer 1, there are a couple of ways that a change in LGN localization from apical to basolateral could be achieved. In embryonic skin, Insc has been shown to recruit LGN apically to promote perpendicular divisions (Williams et al, 2014). In adult tissue however, single-cell RNA-sequencing evidence suggests that, unlike LGN, Insc is not expressed in proliferating basal cells (Reviewer Figure 1) (Joost et al., 2020). In the absence of Insc, there may not be a way for LGN to be recruited to the apical domain. Conversely, SAPCD2, which has been shown to exclude LGN from the apical domain in mouse retinal progenitor cells (Chiu et al., 2016), is expressed in dividing basal cells of the adult epidermis (Reviewer Figure 1) (Joost et al., 2020). If

SAPCD2 carries out a similar function in epidermal cells, it provides a mechanism for ensuring LGN localization at the basolateral cortex.

7) In the "Morrow et al., 2019" paper cited in the manuscript, loss of NuMA leads to more planar divisions that promote tumorigenesis by Kras mutant in skin, which is opposite to what the authors observed: more perpendicular, not planar divisions, promote tumorigenesis. The authors said in the discussion that this happens through "local accumulation of suprabasal cells". It is unclear how accumulation of differentiated cells would accelerate tumor development in the sense of pushing transitioning of papilloma to carcinoma. Please expand on this in the discussion.

We have provided additional data (revised Figure 4, F and G) and updated the discussion to more clearly describe how loss of LGN could accelerate tumor formation (highlighted, pp 12-13).

While deletion of PTEN in the basal layer leads to tumor growth in the snout epidermis, the delay between loss of PTEN and tumor initiation is highly variable. Suzuki et al report a 5-month range in timing of epidermal tumor initiation. Although we have not conducted long-term studies, we have observed a similar variability in the timing of tumor initiation, with some LGN-/- mice forming tumors as early as 15 days post-tamoxifen injections and others were kept for up to 2 months without any visible tumor growth on the snout. This considerable temporal variability suggests that while the PTEN-deficient epidermis is primed for tumor initiation, additional, more rare (local) events or changes are likely needed to kick-start tumor initiation. We hypothesize that spindle orientation defects increase the probability of these events/changes occurring in the tissue, which then translates to an acceleration of tumor initiation. In addition, since about a third of PTEN LGN -/- mice develop snout tumors by 28d, we do not expect to see changes in all of these mice at 14d. Rather, we expect a subset of these mice to exhibit changes at 14d and go on to develop tumors early.

To further address changes that could promote tumor initiation, we took a closer look at the 14d epidermis and found rare proliferative cells (Ki67+) that do not express Krt5 in the suprabasal layer of the epidermis. Furthermore, we observed a subset of mice in the PTEN LGN -/- group with an elevated proportion of these Krt5- Ki67+ cells. To check whether these aberrant cells could be associated with tumor initiation, we analyzed tissue from 28d tumors and found a higher proportion of Krt5- Ki67+ cells, confirming the association. We propose that misoriented divisions give rise to suprabasal cells, which do not, or cannot reintegrate the basal layer and retain the ability to proliferate, contributing to tumor initiation. The thickened basal layer in PTEN-deficient epidermis may prevent suprabasally-positioned cells from making contact with the basement membrane and reintegrating their layer. We were not able to test this hypothesis, as we don't have the mouse lines necessary to perform this experiment (Krt5^{CreERT2}; PTEN$^{fl/fl}$; LGN+/+ or LGN-/- combined with R26^{tdTomato}; H2B-GFP). The fact that these cells appear more frequently in a third of PTEN LGN-/- animals at 14d is consistent with our data showing that one third of mice have accelerated tumor formation and supports our hypothesis that only a subset of animals undergo the changes associated to tumor initiation at that stage.

Finally, we wish to emphasize that this study did not investigate whether LGN impacts the transition from papilloma to carcinoma. Although Suzuki et al. (2003) mentions that tumors occurring in PTEN-deficient do transition to carcinomas, the timing of this transition has not been well described. Because of lethality in LGN- and PTEN-deficient animals past 28d, likely due to problems in other K5-expressing tissues such as the esophagus, we could not keep the animals longer to assess the potential effects of LGN on this transition.

References

Cetera, M., L. Leybova, B. Joyce, and D. Devenport. 2018. Counter-rotational cell flows drive morphological and cell fate asymmetries in mammalian hair follicles. *Nat Cell Biol*. 20:541-552.

Chiu, C.W.N., C. Monat, M. Robitaille, M. Lacomme, A.M. Daulat, G. Macleod, H. McNeill, M. Cayouette, and S. Angers. 2016. SAPCD2 Controls Spindle Orientation and Asymmetric Divisions by Negatively Regulating the Galphai-LGN-NuMA Ternary Complex. *Dev Cell*. 36:50-62.
Choi, B.H., S. Xie, and W. Dai. 2017. PTEN is a negative regulator of mitotic checkpoint complex during the cell cycle. *Exp Hematol Oncol*. 6:19.
Ipponjima, S., T. Hibi, and T. Nemoto. 2016. Three-Dimensional Analysis of Cell Division Orientation in Epidermal Basal Layer Using Intravital Two-Photon Microscopy. *PLoS One*. 11:e0163199.
Joost, S., K. Annusver, T. Jacob, X. Sun, T. Dalessandri, U. Sivan, I. Sequeira, R. Sandberg, and M. Kasper. 2020. The Molecular Anatomy of Mouse Skin during Hair Growth and Rest. *Cell Stem Cell*. 26:441-457.e447.
Liu, Y., X. Du, S. Zhang, Y. Liu, Q. Zhang, Q. Yin, M.A. McNutt, and Y. Yin. 2017. PTEN regulates spindle assembly checkpoint timing through MAD1 in interphase. *Oncotarget*. 8:98040-98050.
Lough, K.J., K.M. Byrd, C.P. Descovich, D.C. Spitzer, A.J. Bergman, G.M.r. Beaudoin, L.F. Reichardt, and S.E. Williams. 2019. Telophase correction refines division orientation in stratified epithelia. *Elife*. 8.
Morrow, A., J. Underwood, L. Seldin, T. Hinnant, and T. Lechler. 2019. Regulated spindle orientation buffers tissue growth in the epidermis. *Elife*. 8.
Smart, I.H.M. 1970. Variation in the plane of cell cleavage during the process of stratification in the mouse epidermis. *British Journal of Dermatology*. 82:276-282.
Suzuki, A., S. Itami, M. Ohishi, K. Hamada, T. Inoue, N. Komazawa, H. Senoo, T. Sasaki, J. Takeda, M. Manabe, T.W. Mak, and T. Nakano. 2003. Keratinocyte-specific Pten deficiency results in epidermal hyperplasia, accelerated hair follicle morphogenesis and tumor formation. *Cancer Res*. 63:674-681.
Williams, S.E., S. Beronja, H.A. Pasolli, and E. Fuchs. 2011. Asymmetric cell divisions promote Notch-dependent epidermal differentiation. *Nature*. 470:353-358.
Williams, S.E., L.A. Ratliff, M.P. Postiglione, J.A. Knoblich, and E. Fuchs. 2014. Par3-mInsc and Galphai3 cooperate to promote oriented epidermal cell divisions through LGN. *Nat Cell Biol*. 16:758-769.

RE: Manuscript #E23-03-0111R
TITLE: "LGN loss randomizes spindle orientation and accelerates tumorigenesis in PTEN-deficient epidermis"

Dear Dr. Viala:

Thank you sending this revised manuscript to MBoC. The reviewers were generally more favorable about the manuscript. There were still some issues and will delineate the required changes below. That said, I expect that the changes can be easily made and I will judge revisions by myself (no further review).

1. Minor revisions asked by reviewer #1 and #2.
2. Reviewer #2, point 1. Replicates and stats are required.
3. Reviewer #2, point 2. Demonstration of knockdown efficiency would very much strengthen the conclusions.
4. Reviewer #2, point 3. I think this can be addressed by changes to the text. Please ensure to not overstate conclusions.

Sincerely,
Terry Lechler
Monitoring Editor
Molecular Biology of the Cell

Dear Dr. Viala,

The review of your manuscript, referenced above, is now complete. The Monitoring Editor has decided that your manuscript requires minor revisions before it can be published in Molecular Biology of the Cell, as described in the Monitoring Editor's decision letter above and the reviewer comments (if any) below.

A reminder: Please do not contact the Monitoring Editor directly regarding your manuscript. If you have any questions regarding the review process or the decision, please contact the MBoC Editorial Office (mboc@ascb.org).

When submitting your revision include a rebuttal letter that details, point-by-point, how the Monitoring Editor's and reviewers' comments have been addressed. (The file type for this letter must be "rebuttal letter"; do not include your response to the Monitoring Editor and reviewers in a "cover letter.") Please bear in mind that your rebuttal letter will be published with your paper if it is accepted, unless you have opted out of publishing the review history.

Authors are allowed 15 days to submit a revision. If this time period is inadequate, please contact us immediately at mboc@ascb.org.

In preparing your revised manuscript, please follow the instruction in the Information for Authors (www.molbiolcell.org/info-for-authors). In particular, to prepare for the possible acceptance of your revised manuscript, submit final, publication-quality figures with your revision as described.

To submit the rebuttal letter, revised version, and figures, please use this link (please enable cookies, or cut and paste URL):
Link Not Available

MBoC offers the option to publish your work with immediate open access. Open access can increase the discoverability and usability of your research. If you would like to publish your paper with immediate open access but did not select this option during initial submission, please contact us at mbc@ascb.org..

Authors of Articles and Brief Communications whose manuscripts have returned for minor revision ("revise only") are encouraged to create a short video abstract to accompany their article when it is published. These video abstracts, known as Science Sketches, are up to 2 minutes long and will be published on YouTube and then embedded in the article abstract. Science Sketch Editors on the MBoC Editorial Board will provide guidance as you prepare your video. Information about how to prepare and submit a video abstract is available at www.molbiolcell.org/science-sketches. Please contact mboc@ascb.org if you are interested in creating a Science Sketch.

Thank you for submitting your manuscript to MBoC. Please do not hesitate to contact this office if you have any questions.

Sincerely,

Eric Baker
Journal Production Manager
MBoC Editorial Office
mbc@ascb.org

Reviewer #1 (Remarks to the Author):

The authors have satisfied the main concerns of this reviewer in their revised manuscript.

Minor change requests: authors should consider citing the following key studies demonstrating epithelial cell reintegration phenomenon:

1) Bergstralh DT, Lovegrove HE, St Johnston D. Lateral adhesion drives reintegration of misplaced cells into epithelial monolayers. Nat Cell Biol. 2015 Nov;17(11):1497-1503. doi: 10.1038/ncb3248. Epub 2015 Sep 28. PMID: 26414404; PMCID: PMC4878657.

2) Pfannenstein A, Macara IG. A junction-dependent mechanism drives murine mammary cell intercalation for ductal elongation. Dev Cell. 2023 Jul 10;58(13):1126-1138.e4. doi: 10.1016/j.devcel.2023.04.009. Epub 2023 May 3. PMID: 37141887; PMCID: PMC10524519.

Title for Results section: "Cells positioned suprabasally following oblique divisions can move to realign with the basal daughter cell" is confusing - should be reworded to specify "reintegration"

Line in last results section: "These results indicate that loss of LGN and the concomitant increase in perpendicular and oblique divisions does not affect differentiation or apoptosis, but does affect mitosis."...should be rephrased to suggest increase in "proliferation", since authors do not demonstrate that mitotic integrity is impacted.

Reviewer #2 (Remarks to the Author):

The authors have addressed some of our points. Yet, several concerns remain:
1) Figure 1I and in Figure S3, 28d PTEN LGN+/+ condition shows data derived from a single animal (N = 1), and is therefore of limited use. Please increase the sample size.
2) Uncertainty in how efficient PTEN knockout is. It is necessary to validate that PTEN is knocked out to attribute the observed phenotype between PTEN-/- and PTEN +/+ condition to loss of PTEN (e.g., not because of tamoxifen treatment). It is surprising that the authors are experiencing difficulty isolating epithelial cells from the snout, but alternatively the authors could check basal cells from other anatomic regions (e.g., head or back skin).
3) The new observation of Ki67+ spinous cells (Figure 4F and G) does not explain how the spindle orientation defect towards perpendicular division promotes tumor initiation for the following reasons:
a) One should exercise caution in assigning biological significance to outliers. It would be helpful if the authors could specify why they choose this particular outlier test and if it is suitable for small sample size (N=6 in this case).
b) It is unconvincing to link this observation to tumor initiation simply because the two events are occurring at similar frequency. To make that correlation one need to, for example follow the same mice temporally (e.g. through intravital imaging) and show that proliferating suprabasal cells could only be observed in mice that later develop tumors. The authors could also consider comparing tumor free snout regions from tumor bearing animals with snout regions from animals without tumors and see if the former has more Ki67+ spinous cells, assuming that there are some biological traits of those 1/3rd of animals that make them tumor-prone.
c) Correlation does not equal causation; the proliferating spinous cells could be a phenotype accompanying tumor initiation. In figure 4F it looks like the LGN-/- condition has more Ki67+ basal cells as well, so the spinous Ki67+ might be a phenomenon accompanying regions of skin with more proliferations. The authors could present it as an interesting observation but should not present it as a cause for increased tumor initiation.
Minor comments:
1) Please specify how tumor size/area in Figure 1G and Figure S1C was calculated in the method section. Related, why did the author use volume (mm3) for snout tumors and area (mm2) for eyelid tumors?
2) In some figures it wasn't obvious that the authors were looking only at snout tissue. It would be helpful to clarify this in figure legends or in method section.

Reviewer #1 (Remarks to the Author):

The authors have satisfied the main concerns of this reviewer in their revised manuscript.

Minor change requests: authors should consider citing the following key studies demonstrating epithelial cell reintegration phenomenon:

1) Bergstralh DT, Lovegrove HE, St Johnston D. Lateral adhesion drives reintegration of misplaced cells into epithelial monolayers. Nat Cell Biol. 2015 Nov;17(11):1497-1503. doi: 10.1038/ncb3248. Epub 2015 Sep 28. PMID: 26414404; PMCID: PMC4878657.

2) Pfannenstein A, Macara IG. A junction-dependent mechanism drives murine mammary cell intercalation for ductal elongation. Dev Cell. 2023 Jul 10;58(13):1126-1138.e4. doi: 10.1016/j.devcel.2023.04.009. Epub 2023 May 3. PMID: 37141887; PMCID: PMC10524519.

We added the references (p.9).

Title for Results section: "Cells positioned suprabasally following oblique divisions can move to realign with the basal daughter cell" is confusing - should be reworded to specify "reintegration"

The text was changed to "Cells positioned suprabasally following oblique divisions can **reintegrate into the basal layer"** (p.8).

Line in last results section: "These results indicate that loss of LGN and the concomitant increase in perpendicular and oblique divisions does not affect differentiation or apoptosis, but does affect mitosis."...should be rephrased to suggest increase in "proliferation", since authors do not demonstrate that mitotic integrity is impacted.

We changed the text to: "These results indicate that loss of LGN and the concomitant increase in perpendicular and oblique divisions does not affect differentiation or apoptosis, but does affect **proliferation**." (p.11).

Reviewer #2 (Remarks to the Author):

The authors have addressed some of our points. Yet, several concerns remain:
1) Figure 1I and in Figure S3, 28d PTEN LGN+/+ condition shows data derived from a single animal (N = 1), and is therefore of limited use. Please increase the sample size.

We added the data from the second tumor to both graphs.

2) Uncertainty in how efficient PTEN knockout is. It is necessary to validate that PTEN is knocked out to attribute the observed phenotype between PTEN-/- and PTEN +/+ condition to loss of PTEN (e.g., not because of tamoxifen treatment). It is surprising that the authors are experiencing difficulty isolating epithelial cells from the snout, but alternatively the authors could check basal cells from other anatomic regions (e.g., head or back skin).

We added genotyping data for both the floxed and deleted PTEN alleles in uninduced (mock-injected) and induced (tamoxifen injected) epidermis (revised Figure S1A). We collected footpad epidermis 21d after injection, sorted the basal cells and genotyped them using PCR. It confirmed

that PTEN is deleted upon tamoxifen administration in most basal cells for both groups. Changes to the text were made on p.4 and a methods section was added on p.14.

3) The new observation of Ki67+ spinous cells (Figure 4F and G) does not explain how the spindle orientation defect towards perpendicular division promotes tumor initiation for the following reasons:
a) One should exercise caution in assigning biological significance to outliers. It would be helpful if the authors could specify why they choose this particular outlier test and if it is suitable for small sample size (N=6 in this case).
b) It is unconvincing to link this observation to tumor initiation simply because the two events are occurring at similar frequency. To make that correlation one need to, for example follow the same mice temporally (e.g. through intravital imaging) and show that proliferating suprabasal cells could only be observed in mice that later develop tumors. The authors could also consider comparing tumor free snout regions from tumor bearing animals with snout regions from animals without tumors and see if the former has more Ki67+ spinous cells, assuming that there are some biological traits of those 1/3rd of animals that make them tumor-prone.
c) Correlation does not equal causation; the proliferating spinous cells could be a phenotype accompanying tumor initiation. In figure 4F it looks like the LGN-/- condition has more Ki67+ basal cells as well, so the spinous Ki67+ might be a phenomenon accompanying regions of skin with more proliferations. The authors could present it as an interesting observation but should not present it as a cause for increased tumor initiation.

We made changes to the text to ensure that we are not overstating conclusions (p. 13).

Minor comments:
1) Please specify how tumor size/area in Figure 1G and Figure S1C was calculated in the method section. Related, why did the author use volume (mm3) for snout tumors and area (mm2) for eyelid tumors?

Tumor size was calculated using measurements made from pictures of the mice taken from different angles. While the pictures were sufficient to estimate the volume of tumours on the snout, because of the smaller size and localization of eyelid tumors, we were restricted to estimating the area of tumours. This has been added to the methods section (p.14).

2) In some figures it wasn't obvious that the authors were looking only at snout tissue. It would be helpful to clarify this in figure legends or in method section.

The information was added in the legend title of Figure 3.

RE: Manuscript #E23-03-0111RR
TITLE: "LGN loss randomizes spindle orientation and accelerates tumorigenesis in PTEN-deficient epidermis"

Dear Dr. Viala:

I am pleased to accept your manuscript for publication in Molecular Biology of the Cell.

Congratulations and thank you for submitting this work.

Sincerely,
Terry Lechler
Monitoring Editor
Molecular Biology of the Cell

Dear Dr. Viala:

Congratulations on the acceptance of your manuscript! Thank you for choosing to publish your work in Molecular Biology of the Cell (MBoC).

Within 10 days, an unedited PDF of your manuscript will be published on MBoC in Press, an early release version of the journal. The date your manuscript appears on this site is the official publication date.

Your copyedited and typeset manuscript will be scheduled for publication in the next available issue of MBoC. Our production team will notify you when the page proofs of your paper are ready for your review.

MBoC offers the option to publish your paper with immediate open access. Open access can increase the discoverability and usability of your research. If you would like to publish your paper with immediate open access but did not select this option during initial submission, please contact the MBoC Editorial Office (mbc@ascb.org).

We invite you to submit images related to your accepted manuscript to be considered for the journal cover. Please contact mboc@ascb.org to learn how to submit candidate cover images.

Authors of Articles and Brief Communications are also encouraged to create a short video abstract to accompany their article when it is published. These video abstracts, known as Science Sketches, are up to 2 minutes long and will be published on YouTube and then embedded in the article abstract. Science Sketch Editors on the MBoC Editorial Board will provide guidance as you prepare your video. Information about how to prepare and submit a video abstract is available at www.molbiolcell.org/science-sketches. Please contact mboc@ascb.org if you are interested in creating a Science Sketch.

We look forward to publishing your paper in MBoC.
Sign up for MBoC's new content alerts here https://tinyurl.com/3bcsyk3e

Sincerely,

Eric Baker
Journal Production Manager
MBoC Editorial Office
mbc@ascb.org
